# Author Correction: Ongoing declines for the world’s amphibians in the face of emerging threats

**DOI:** 10.1038/s41586-023-06851-6

**Published:** 2023-12-01

**Authors:** Jennifer A. Luedtke, Janice Chanson, Kelsey Neam, Louise Hobin, Adriano O. Maciel, Alessandro Catenazzi, Amaël Borzée, Amir Hamidy, Anchalee Aowphol, Anderson Jean, Ángel Sosa-Bartuano, Ansel Fong G., Anslem de Silva, Antoine Fouquet, Ariadne Angulo, Artem A. Kidov, Arturo Muñoz Saravia, Arvin C. Diesmos, Atsushi Tominaga, Biraj Shrestha, Brian Gratwicke, Burhan Tjaturadi, Carlos C. Martínez Rivera, Carlos R. Vásquez Almazán, Celsa Señaris, S. R. Chandramouli, Christine Strüssmann, Claudia Fabiola Cortez Fernández, Claudio Azat, Conrad J. Hoskin, Craig Hilton-Taylor, Damion L. Whyte, David J. Gower, Deanna H. Olson, Diego F. Cisneros-Heredia, Diego José Santana, Elizah Nagombi, Elnaz Najafi-Majd, Evan S. H. Quah, Federico Bolaños, Feng Xie, Francisco Brusquetti, Francisco S. Álvarez, Franco Andreone, Frank Glaw, Franklin Enrique Castañeda, Fred Kraus, Gabriela Parra-Olea, Gerardo Chaves, Guido F. Medina-Rangel, Gustavo González-Durán, H. Mauricio Ortega-Andrade, Iberê F. Machado, Indraneil Das, Iuri Ribeiro Dias, J. Nicolas Urbina-Cardona, Jelka Crnobrnja-Isailović, Jian-Huan Yang, Jiang Jianping, Jigme Tshelthrim Wangyal, Jodi J. L. Rowley, John Measey, Karthikeyan Vasudevan, Kin Onn Chan, Kotambylu Vasudeva Gururaja, Kristiina Ovaska, Lauren C. Warr, Luis Canseco-Márquez, Luís Felipe Toledo, Luis M. Díaz, M. Monirul H. Khan, Madhava Meegaskumbura, Manuel E. Acevedo, Marcelo Felgueiras Napoli, Marcos A. Ponce, Marcos Vaira, Margarita Lampo, Mario H. Yánez-Muñoz, Mark D. Scherz, Mark-Oliver Rödel, Masafumi Matsui, Maxon Fildor, Mirza D. Kusrini, Mohammad Firoz Ahmed, Muhammad Rais, N’Goran G. Kouamé, Nieves García, Nono Legrand Gonwouo, Patricia A. Burrowes, Paul Y. Imbun, Philipp Wagner, Philippe J. R. Kok, Rafael L. Joglar, Renoir J. Auguste, Reuber Albuquerque Brandão, Roberto Ibáñez, Rudolf von May, S. Blair Hedges, S. D. Biju, S. R. Ganesh, Sally Wren, Sandeep Das, Sandra V. Flechas, Sara L. Ashpole, Silvia J. Robleto-Hernández, Simon P. Loader, Sixto J. Incháustegui, Sonali Garg, Somphouthone Phimmachak, Stephen J. Richards, Tahar Slimani, Tamara Osborne-Naikatini, Tatianne P. F. Abreu-Jardim, Thais H. Condez, Thiago R. De Carvalho, Timothy P. Cutajar, Todd W. Pierson, Truong Q. Nguyen, Uğur Kaya, Zhiyong Yuan, Barney Long, Penny Langhammer, Simon N. Stuart

**Affiliations:** 1 Re:wild, Austin, TX USA; 2 IUCN SSC Amphibian Specialist Group, Toronto, Ontario Canada; 3 Museu Paraense Emílio Goeldi, CZO/Herpetologia, Belém, Brazil; 4 Florida International University, Miami, FL USA; 5 Centro de Ornitologia y Biodiversidad (CORBIDI), Lima, Peru; 6 Laboratory of Animal Behaviour and Conservation, College of Life Sciences, Nanjing Forestry University, Nanjing, People’s Republic of China; 7 Laboratory of Herpetology, Museum Zoologicum Bogoriense, Research Center for Biosystematics and Evolution, National Research and Innovation Agency (BRIN), Cibinong, Indonesia; 8 Department of Zoology, Faculty of Science, Kasetsart University, Bangkok, Thailand; 9 Action Pour la Sauvegarde de l’Ecologie en Haïti (ACSEH), Les Cayes, Haiti; 10 Environmental Protection In the Caribbean (EPIC), Maho, Sint Maarten; 11 Museo de Vertebrados de la Universidad de Panamá, Ciudad de Panama, Panama; 12 Centro Oriental de Ecosistemas y Biodiversidad (BIOECO), Museo de Historia Natural “Tomás Romay”, Santiago de Cuba, Cuba; 13 IUCN SSC Amphibian Specialist Group, Sri Lanka, Gampola, Sri Lanka; 14 Laboratoire Évolution & Diversité Biologique, UMR 5174, Université Toulouse III Paul Sabatier, Toulouse, France; 15 Russian State Agrarian University—MTAA, Moscow, Russia; 16 IUCN SSC Amphibian Specialist Group Bolivia, La Paz, Bolivia; 17 Animal Nutrition Unit, Department of Veterinary and Biosciences, Ghent University, Ghent, Belgium; 18 ASEAN Centre for Biodiversity, University of the Philippines Los Baños, Laguna, Philippines; 19 HerpWatch Pilipinas, Manila, Philippines; 20 Faculty of Education, University of the Ryukyus, Okinawa, Japan; 21 Graduate School of Engineering and Science, University of the Ryukyus, Okinawa, Japan; 22 SAVE THE FROGS!, Laguna Beach, CA USA; 23 The University of Texas at Arlington, Arlington, TX USA; 24 Smithsonian Conservation Biology Institute, Front Royal, VA USA; 25 Center for Environmental Studies, Sanata Dharma University (CESSDU), Yogyakarta, Indonesia; 26 Pinelands Preservation Alliance, Southampton Township, NJ USA; 27 Centro de Conservación de Anfibios, Amaru Bioparque, Cuenca, Ecuador; 28 Museo de Historia Natural, Escuela de Biologia, Universidad de San Carlos, Guatemala City, Guatemala; 29 FUNDAECO, Guatemala City, Guatemala; 30 Estación Biológica de Doñana (EBD-CSIC), Seville, Spain; 31 Department of Ecology and Environmental Sciences, Pondicherry University, Puducherry, India; 32 Universidade Federal de Mato Grosso, Cuiabá, Brazil; 33 Museo Nacional de Historia Natural, La Paz, Bolivia; 34 Sustainability Research Center & PhD Program in Conservation Medicine, Faculty of Life Sciences, Universidad Andres Bello, Santiago, Chile; 35 College of Science & Engineering, James Cook University, Townsville, Queensland Australia; 36 David Attenborough Building, IUCN, Cambridge, UK; 37 Department of Life Sciences, University of the West Indies Mona, Kingston, Jamaica; 38 The Natural History Museum, London, UK; 39 Pacific Northwest Research Station, United States Department of Agriculture, Forest Service, Corvallis, OR USA; 40 Universidad San Francisco de Quito USFQ, Colegio de Ciencias Biológicas y Ambientales, Instituto de Biodiversidad Tropical IBIOTROP, Quito, Ecuador; 41 Instituto Nacional de Biodiversidad INABIO, Quito, Ecuador; 42 Instituto de Biociências, Universidade Federal de Mato Grosso do Sul, Campo Grande, Brazil; 43 The New Guinea Binatang Research Center, Madang, Papua New Guinea; 44 Department of Zoology, Faculty of Science, Ege University, İzmir, Turkey; 45 Institute for Tropical Biology and Conservation, Universiti Malaysia Sabah, Kota Kinabalu, Malaysia; 46 Lee Kong Chian Natural History Museum, National University of Singapore, Singapore, Singapore; 47 Escuela de Biología, Universidad de Costa Rica, San José, Costa Rica; 48 CIBET (Museo de Zoología), Universidad de Costa Rica, San José, Costa Rica; 49 Chengdu Institute of Biology, Chinese Academy of Sciences, Chengdu, People’s Republic of China; 50 Instituto de Investigación Biológica del Paraguay, Asunción, Paraguay; 51 Fundación Naturaleza El Salvador, San Salvador, El Salvador; 52 Museo Regionale di Scienze Naturali, Torino, Italy; 53 Zoologische Staatssammlung München (ZSM-SNSB), Munich, Germany; 54 Panthera, Tegucigalpa, Honduras; 55 Department of Ecology and Evolutionary Biology, University of Michigan, Ann Arbor, MI USA; 56 Instituto de Biologia, Universidad Nacional Autónoma de México, Mexico City, Mexico; 57 Instituto de Ciencias Naturales, Universidad Nacional de Colombia, Bogotá D.C., Colombia; 58 WCS-Colombia, Cali, Colombia; 59 Biogeography and Spatial Ecology Research Group, Life Sciences Faculty, Universidad Regional Amazónica IKIAM, Tena, Ecuador; 60 Herpetology Division, Instituto Nacional de Biodiversidad, Quito, Ecuador; 61 Instituto Boitatá de Etnobiologia e Conservação da Fauna, Goiânia, Brazil; 62 Institute of Biodiversity and Environmental Conservation, Universiti Malaysia Sarawak, Kota Samarahan, Malaysia; 63 Departamento de Ciências Biológicas, Universidade Estadual de Santa Cruz, Ilhéus, Brazil; 64 Departamento de Ecología y Territorio, Facultad de Estudios Ambientales y Rurales, Pontificia Universidad Javeriana, Bogotá, Colombia; 65 Department of Biology and Ecology, Faculty of Sciences and Mathematics, University of Niš, Niš, Serbia; 66 Kadoorie Farm and Botanic Garden, Hong Kong SAR, People’s Republic of China; 67 University of New England, Armidale, New South Wales Australia; 68 Bhutan Ecological Society, Thimphu, Bhutan; 69 Australian Museum Research Institute, Australian Museum, Sydney, New South Wales Australia; 70 Centre for Ecosystem Science, School of Biological, Earth and Environmental Sciences (BEES), University of New South Wales, Sydney, New South Wales Australia; 71 Centre for Invasion Biology, Department of Botany & Zoology, Stellenbosch University, Stellenbosch, South Africa; 72 Centre for Invasion Biology, Institute of Biodiversity, School of Ecology and Environmental Science, Yunnan University, Kunming, People’s Republic of China; 73 Laboratory for the Conservation of Endangered Species, CSIR-Centre for Cellular and Molecular Biology, Hyderabad, India; 74 Srishti Manipal Institute of Art, Design and Technology, Manipal Academy of Higher Education, Manipal, India; 75 Biolinx Environmental Research, Victoria, British Columbia Canada; 76 Royal British Columbia Museum, Victoria, British Columbia Canada; 77 Flint, TX USA; 78 Laboratorio de Herpetología, Facultad de Ciencias, Universidad Nacional Autónoma de México, Mexico City, Mexico; 79 Laboratório de História Natural de Anfíbios Brasileiros (LaHNAB), Universidade Estadual de Campinas (Unicamp), São Paulo, Brazil; 80 Museo Nacional de Historia Natural de Cuba, La Habana, Cuba; 81 Department of Zoology, Jahangirnagar University, Dhaka, Bangladesh; 82 Key Laboratory in Forest Ecology and Conservation, College of Forestry, Guangxi University, Nanning, People’s Republic of China; 83 Museo Nacional de Historia Natural “Jorge A. Ibarra”, Ciudad de Guatemala, Guatemala; 84 Instituto de Biologia, Campus Universitário de Ondina, Universidade Federal da Bahia, Salvador, Brazil; 85 David, Panama; 86 Instituto de Ecorregiones Andinas (INECOA, UNJu—Conicet), San Salvador de Jujuy, Argentina; 87 Instituto Venezolano de Investigaciones Científicas (IVIC), Miranda, Venezuela; 88 Fundación para el Desarrollo de las Ciencias Físicas, Matemáticas y Naturales (FUDECI), Caracas, Venezuela; 89 Unidad de Investigación, Instituto Nacional de Biodiversidad (INABIO), Quito, Ecuador; 90 Natural History Museum of Denmark, University of Copenhagen, Copenhagen, Denmark; 91 Museum für Naturkunde—Leibniz Institute for Evolution and Biodiversity Science, Berlin, Germany; 92 Kyoto University, Yoshida Nihonmatsu, Kyoto, Japan; 93 Faculty of Forestry & Environment, IPB University, Bogor, Indonesia; 94 Aaranyak, Guwahati, India; 95 Herpetology Lab, Department of Zoology, Wildlife and Fisheries, Pir Mehr Ali Shah Arid Agriculture University Rawalpindi, Rawalpindi, Pakistan; 96 Laboratoire de Biodiversité et Ecologie Tropicale, UFR Environnement, Université Jean Lorougnon Guédé, Daloa, Côte d’Ivoire; 97 IUCN Species Survival Commission, Gland, Switzerland; 98 Laboratory of Zoology, Faculty of Science, University of Yaoundé I, Yaoundé, Cameroon; 99 Department of Biology, University of Puerto Rico, San Juan, Puerto Rico; 100 Zoology Unit, Research and Education Section, Sabah Parks, Kota Kinabalu, Malaysia; 101 Allwetterzoo, Münster, Germany; 102 Center for Biodiversity and Ecosystem, Villanova University, Villanova, PA USA; 103 Department of Ecology and Vertebrate Zoology, Faculty of Biology and Environmental Protection, University of Łódź, Łódź, Poland; 104 Department of Life Sciences, The Natural History Museum, London, UK; 105 Rio Piedras Campus, University of Puerto Rico, San Juan, Puerto Rico; 106 Proyecto Coqui, San Juan, Puerto Rico; 107 Department of Life Sciences, The University of the West Indies, St Augustine, Trinidad and Tobago; 108 Laboratório de Fauna e Unidades de Conservação, Universidade de Brasília, Brasília-DF, Brazil; 109 Smithsonian Tropical Research Institute, Panama, República de Panamá; 110 California State University Channel Islands, Camarillo, CA USA; 111 Center for Biodiversity, Temple University, Philadelphia, PA USA; 112 Systematics Lab, Department of Environmental Studies, University of Delhi, Delhi, India; 113 Chennai Snake Park, Chennai, India; 114 Department of Zoology, University of Otago, Dunedin, New Zealand; 115 Centre for Research in Emerging Tropical Diseases, Department of Zoology, University of Calicut, Kerala, India; 116 EDGE of Existence programme, Conservation and Policy, Zoological Society of London, London, UK; 117 Bichos.team, Bogotá, Colombia; 118 Environmental Studies, St Lawrence University, Canton, NY USA; 119 Prescott, Ontario Canada; 120 IUCN SSC Amphibian Specialist Group Nicaragua, Managua, Nicaragua; 121 Grupo Jaragua, Santo Domingo, Dominican Republic; 122 Department of Organismic and Evolutionary Biology and Museum of Comparative Zoology, Harvard University, Cambridge, MA USA; 123 Department of Biology, Faculty of Natural Sciences, National University of Laos, Vientiane, Laos; 124 Herpetology Department, South Australian Museum, Adelaide, South Australia Australia; 125 Faculty of Sciences Sremlalia, Cadi Ayyad University, Marrakech, Morocco; 126 School of Agriculture, Geography, Environment, Ocean and Natural Sciences, The University of the South Pacific, Suva, Fiji; 127 Department of Earth Sciences, Carleton University, Ottawa, Ontario Canada; 128 Universidade Federal de Minas Gerais, Belo Horizonte, Brazil; 129 Department of Ecology, Evolution and Organismal Biology, Kennesaw State University, Kennesaw, GA USA; 130 Institute of Ecology and Biological Resources, Vietnam Academy of Science and Technology, Ha Noi, Viet Nam; 131 School of Life Sciences, Southwest University, Chongqing, People’s Republic of China; 132 Arizona State University, Tempe, AZ USA; 133 A Rocha International, London, UK; 134 Synchronicity Earth, London, UK

**Keywords:** Biodiversity, Conservation biology, Biodiversity, Conservation biology

Correction to: *Nature* 10.1038/s41586-023-06578-4 Published online 4 October 2023

In the version of the article initially published, the first name of Somphouthone Phimmachak appeared incorrectly as Soumphthone. The Data Availability section included the wrong link, which has been updated to https://www.iucnredlist.org/resources/data-repository. These changes have been made in the HTML and PDF versions of the article.

