# Supplementary material for: Ongoing declines for the world’s amphibians in the face of emerging threats
Source: Nature. 2023 Oct 4;622(7982):308–14. doi: 10.1038/s41586-023-06578-4 (PMC10567568; doi:10.1038/s41586-023-06578-4)
Supplement: Supplementary file 1 — Supplementary Notes 1–3 and Supplementary Tables 1–4. [file 41586_2023_6578_MOESM1_ESM.pdf]

nature portfolio

https://doi.org/10.1038/s41586-023-06578-4

Supplementary information

Ongoing declines for the world's amphibians in the face of emerging threats

In the format provided by the authors and unedited

Supplementary Information for

Ongoing declines for the world's amphibians in the face of emerging threats

Jennifer A. Luedtke, Janice Chanson, Kelsey Neam, Louise Hobin, Adriano O. Maciel, Alessandro Catenazzi, Amaël Borzée, Amir Hamidy, Anchalee Aowphol, Anderson Jean, Ángel Sosa-Bartuano, Ansel Fong G., Anslem de Silva, Antoine Fouquet, Ariadne Angulo, Artem A. Kidov, Arturo Muñoz Saravia, Arvin C. Diesmos, Atsushi Tominaga, Biraj Shrestha, Brian Gratwicke, Burhan Tjaturadi, Carlos C. Martínez Rivera, Carlos R. Vásquez Almazán, Celsa Señaris, Chandramouli S.R., Christine Strüssmann, Claudia Fabiola Cortez Fernández, Claudio Azat, Conrad J. Hoskin, Craig Hilton-Taylor, Damion L. Whyte, David J. Gower, Deanna H. Olson, Diego F. Cisneros-Heredia, Diego José Santana, Elizah Nagombi, Elnaz Najafi-Majd, Evan S.H. Quah, Federico Bolaños, Feng Xie, Francisco Brusquetti, Francisco S. Álvarez, Franco Andreone, Frank Glaw, Franklin Enrique Castañeda, Fred Kraus, Gabriela Parra-Olea, Gerardo Chaves, Guido F. Medina-Rangel, Gustavo González-Durán, H. Mauricio Ortega-Andrade, Iberê F. Machado, Indraneil Das, Iuri Ribeiro Dias, J. Nicolas Urbina-Cardona, Jelka Crnobrnja-Isailović, Jian-Huan Yang, Jiang Jianping, Jigme Tshelthrim Wangyal, Jodi J.L. Rowley, John Measey, Karthikeyan Vasudevan, Kin Onn Chan, Kotambylu Vasudeva Gururaja, Kristiina Ovaska, Lauren C. Warr, Luis Canseco-Márquez, Luís Felipe Toledo, Luis M. Díaz, M. Monirul H. Khan, Madhava Meegaskumbura, Manuel E. Acevedo, Marcelo Felgueiras Napoli, Marcos A. Ponce, Marcos Vaira, Margarita Lampo, Mario H. Yánez-Muñoz, Mark D. Scherz, Mark-Oliver Rödel, Masafumi Matsui, Maxon Fildor, Mirza D. Kusrini, Mohammad Firoz Ahmed, Muhammad Rais, N'Goran G. Kouamé, Nieves García, Nono Legrand Gonwouo, Patricia A. Burrowes, Paul Y. Imbun, Philipp Wagner, Philippe J. R. Kok, Rafael L. Joglar, Renoir J. Auguste, Reuber Albuquerque Brandão, Roberto Ibáñez, Rudolf von May, S. Blair Hedges, S.D. Biju, S.R. Ganesh, Sally Wren, Sandeep Das, Sandra V. Flechas, Sara L. Ashpole, Silvia J. Robleto-Hernández, Simon P. Loader, Sixto J. Incháustegui, Sonali Garg, Somphouthone Phimmachak, Stephen J. Richards, Tahar Slimani, Tamara Osborne-Naikatini, Tatianne P. F. Abreu-Jardim, Thais H. Condez, Thiago R. De Carvalho, Timothy P. Cutajar, Todd W. Pierson, Truong Q. Nguyen, Ugur Kaya, Zhiyong Yuan, Barney Long, Penny Langhammer, Simon N. Stuart

Correspondence to: jluedtke@rewild.org

This PDF file includes:

Supplementary Notes 1-3
Supplementary Tables 1–4

Supplementary Note 1: Coordination of the GAA2

The Amphibian Red List Authority (ARLA) is the section of the IUCN SSC Amphibian Specialist Group (ASG) responsible for the IUCN Red List assessments of all the world's amphibian species. The ARLA coordinated the GAA2, the second comprehensive assessment of all amphibians.

The ARLA team members at the time of completion of the GAA2 were Jennifer Luedtke (Coordinator), Kelsey Neam, Louise Hobin, and Janice Chanson. Past team members include Nieves García (2020–2021), Lauren Warr (2019–2021), Sean Lyon (2019), Elena Garollo (2015–2016), and Ariadne Angulo (Coordinator from 2009–2014).

The team worked closely with the ASG Regional Chairs, ARLA Regional Coordinators, and many volunteer, intern, or partner organization Facilitators, Compilers, and Reviewers (see Section 4). Regional Chairs and Coordinators advised on the best approach to the assessment process for their region, some coordinated the update process, some planned the consultation workshops, and some served as facilitators and/or data providers. These Regional Coordinators and Chairs are listed in Section 4.1. Some of these individuals changed over the course of the GAA2, which spanned the 2013–2016, 2016–2020, 2021–2025 IUCN quadrennium periods.

The IUCN Red List Unit (RLU) based in Cambridge, UK performed final checks to the supporting documentation of all assessments after they passed independent review. The RLU managed the publication of all assessments on the IUCN Red List website. In addition, Craig Hilton-Taylor provided extensive technical support regarding the use of the IUCN Species Information Service (SIS) online centralized database, and backcasting and Red List Index methods.

The Red List Technical Working Group provided key advice regarding the application of the categories and criteria to complex scenarios involving the threats of *Bd*, *Bsal*, and climate change.

The ARLA advisory group consists of Dr. Simon Stuart (A Rocha International), Dr. Penny Langhammer (Re:wild), Dr. Pria Ghosh (Synchronicity Earth), and Dr. Barney Long (Re:wild).

Supplementary Note 2: Expert Consultation

The following GAA2 workshops were held:

- **West and Central Africa, and East Africa**: Trento, Italy; May 2012.
- **Madagascar**: Ranomafana, Madagascar; 18–22 November 2014 and Munich, Germany; 23–27 November 2015.
- **Mexico**: Mexico City, Mexico; 2014 and Mexico City, Mexico; 21–24 October 2019.
- **West and Central Africa**: Berlin, Germany; 4-8 July 2015.
- **East Africa**: London, UK; 2015–2016.
- **Chile**: Santiago, Chile; 9–10 July 2015.
- **Southern Africa**: South Africa; November 2015.
- **Panama**: Panama City, Panama; 23 October 2015 and 12–16 August 2019.
- **Ecuador**: Cosanga Napo, Ecuador; 16–19 July 2016.
- **Colombia**: Medellín, Colombia; 1–5 August 2016 and Bogotá, Colombia; 27 June–3 July 2018.
- **Peru**: Illinois, USA; 17–21 April 2017.
- **Indonesia**: Bogor, Indonesia; 15–19 May 2017.
- **Philippines**: Tagaytay City, Philippines; 23–26 May 2017.
- **Argentina**: Salta, Argentina; 2–5 October 2017.
- **Guiana Shield**: Brussels, Belgium; 13–16 November 2017.
- **Malaysia**: George Town, Penang, Malaysia; 15–18 January 2018 and Sarawak, Malaysia; 6–9 March 2018.
- **Honduras**: Tegucigalpa, Honduras; 11–15 March 2019.
- **China**: Chengdu, Sichuan, China; 17–21 June 2019.
- **Melanesia**: Port Moresby, Papua New Guinea; 17–20 and 22–25 July 2019 and Brisbane, Australia; 1–4 August 2019.
- **Guatemala and Belize**: Tecpán, Guatemala; 19–22 August 2019.
- **Europe**: Milan; Italy, 2–6 September 2019.
- **Costa Rica**: San José, Costa Rica; 9–13 September 2019.
- **Bolivia**: Cochabamba, Bolivia; 23–27 September 2019.
- **Sri Lanka**: Wattala, Sri Lanka; 17–20 February 2020.
- **Caribbean**: virtual workshop through 50+ online meetings; June–October 2020.
- **Brazil**: virtual workshop through 12 online meetings for the Pampa, Pantanal, Cerrado, Caatinga, Mata Atlântica, Amazônia biomes; December 2020–April 2021.
- **Mainland South Asia**: virtual workshop through 70+ online meetings; August 2020–December 2020.

Due to the emergence of the COVID-19 pandemic, in-person workshops planned for Mainland Southeast Asia, South Asia, and Brazil in 2020 were completed online.

Expert consultation was completed primarily through email and phone correspondence for the following regions:

- Argentina
- Australia
- Canada
- Commonwealth of Independent States and Mongolia

- Japan
- Lesser Antilles
- New Zealand, Fiji, and Palau
- North Africa
- North and South Korea
- Mainland Southeast Asia
- El Salvador
- Paraguay
- Seychelles
- United States
- Uruguay

Part (or all) of the expert consultation was completed through consultant contracts to partner organizations and/or co-authors for the following regions:

- Arabia and the Middle East
- Brazil
- China
- Colombia
- Cuba
- Ecuador
- Mainland Southeast Asia - endemics
- Melanesia
- Nicaragua
- Venezuela
- West and Central Asia

Supplementary Note 3: Extended Acknowledgements

The GAA2 was an extensive collaboration of over 1,000 individuals. The co-authors of this paper acknowledge their contributions and ask their forgiveness for any names inadvertently omitted or misspelled.

ARLA Regional Coordinators

Alessandro Catenazzi, Annika Hillers, Antoine Fouquet, Arvin C. Diesmos, Brian Gratwicke, Dale Roberts, Diego F. Cisneros-Heredia, Feng Xie, Franco Andreone, Geoff Hammerson, Kotambylu Vasudeva Gururaja, Ibere F. Machado, Jelka Crnobrnja-Isailović, Jodi J.L. Rowley, Josiah Townsend, John Measey, Juan Manual Daza, Kin Onn Chan, Madhava Meegaskumbura, Magno Segalla, Marcio Martins, Marcos Vaira, Mauricio Rivera-Correa, Michael Lau, Michele Menegon, Mirza D. Kusrini, N'Goran G. Kouamé, Nono LeGrand Gonwouo, Rachunliu Kamei, Rudolf von May, S. Blair Hedges, Sergius Kusmin, Simon P. Loader, Stephen J. Richards, Uğur Kaya.

ASG Global and Regional Chairs

Allan Gutiérrez, Amir Hamidy, Andres Merino-Viteri, Anslem de Silva, Ariadne Angulo, Artem A. Kidov, Arturo Muñoz Saravia, Arvin C. Diesmos, Carlos R. Vásquez Almazán, Celsa Señaris, Claudia Fabiola Cortez Fernández, Claudio Azat, Cybele Lisboa, Debora Silvano, Deanna H. Olson, Esteban Lavilla, Francisco S. Álvarez, Franco Andreone, Gabriela Parra-Olea, Gerardo Chaves, Giussepe Gagliardi, Jean-Marc Hero, Jeanne Tarrant, Jiang Jianping, Jodi J.L. Rowley, John Measey, Jonathan Kolby, Jose Langone, J. Nicolas Urbina-Cardona, Juan Carlos Ortiz, Karthikeyan Vasudevan, Kin Onn Chan, Kristiina Ovaska, Madhava Meegaskumbura, Margarita Lampo, Mark-Oliver Rödel, Mauricio Rivera-Correa, N'goran Kouamé, Nono LeGrand Gonwouo, Phil Bishop, Philippe J.R. Kok, Pritpal “Micky” Soorae, Roberto Ibáñez, Sandra V. Flechas, Sanjay Molur, Sara L. Ashpole, Silvia J. Robleto-Hernández, Simon P. Loader, Stephen J. Richards, Tahar Slimani, Uğur Kaya, Victor Vargas, Victor Wasonga.

GAA2 Facilitators, Compilers, and Reviewers

Outside of the core ARLA team, the following individuals served as facilitators, compilers, and/or reviewers as a volunteer, paid intern, or from within a partner organization.

Adrian Hoffman, Ahm Reza, Aina Pascuale, Alessandro Catenazzi, Alison Flores, Alyssa Wetterau, Ana Nicole Acosta, Angelica Cervantes, Argelia Rodríguez, Becky Johnson, Ben Tapley, Bruce Young, Casey Green, Catharina Gallacher, Catherine Welsh, Chi Phan, Chris Portway, Collin Van Buren, Corey Roelke, Daniel Absolon, Duncan Sharp, Elizah Nagombi, Emilia Peñaherrera, Emily Strautins, Emmanuel Rivera Téllez, Enzo Carias Perdomo, Erin McGrew, Esther Quintero, Evie Morris, Felix Baier, Freya Wadlow, Gabryella de Sousa Mesquita, Geetha Ortac, Grace Fields, Grace Reyes, Grisel Velásquez, Harry Leung, Hermenegildo Matimele, Hugo Costa, Ingrid Márquez Molina, Jacqueline Mallinson, Jaime García Moreno, Jelka Crnobrnja-Isailović, Jing Zhang, John Lamoreux, Jordann Crawford-Ash, Jorge Rodríguez-Matamoros, José Mario Solís Ramos, Juan Vicente Hernández, Justin Nowakowski, Kathy Potter, Kimberlyn Fonseca, Kumiko Yoneda, Laurence Jarvis, Lucy Coals, Madeline Sheard, Maiko Lutz, Marcelo Tognelli, Marcy Sieggreen, Mariella Superina, Marvin

Tórrez, Milhara Kankanamge, Nadine Samy, Nancy Butler, Neil Cox, Omar Rojas-Padilla, Paul Buzzard, Paulina Arias Caballero, Philip Bowles, Rafael Ramirez, Rob Ward, Roshni Sharma, Ruth Marcec-Greaves, Sean Lyon, Simeon Bezeng, Simon Stuart, Steve Allain, Steve Best, Steven Megson, Tasneem Vaira, Timothy P. Cutajar, Vera Hugues-Salas, Ygrein Roos.

Consultants

The following individuals received contracts from the ARLA or directly from donors to coordinate regional assessment efforts, input data into SIS, and/or facilitate workshops or other assessment processes.

Ana Nicole Acosta, Bruce Young, Eliza Nagombi, Elnaz Najafi-Majd, Fernando Rojas-Runjaic, Gabryella de Sousa, Grace Reyes, Iberê Farina Machado, Celsa Senaris, Jing Zhang, Marcelo Tognelli, Margarita Lampo, Marvin Tórrez, Neil Cox, Phil Bowles, Sheila Pereira de Andrade, Stephen Richards, Tatianne Abreu-Jardin, Timothy Cutajar, Uğur Kaya, Werther Pereira Ramalho, Zhao Yuntao.

Subject-matter experts

The following 1,054 individuals provided information for the IUCN Red List assessments.

Abdulaziz Al-Qahtani, Abel Batista, Abhijit Das, Adam Clause, Adolfo Amézquita, Adrián García Rodríguez, Adrian Antônio Garda, Adriano Oliveira Maciel, Aimee McIntyre, Akshay Gawade, Alan Channing, Albertina Pimentel Lima, Alberto Estrada, Alberto Gosá, Alberto Puente-Rolón, Alberto Sánchez-Vialas, Aldemar Acevedo Rincón, Aldrin Mallari, Alejandro Ramírez, Alejandro Arteaga-Navarro, Alejandro Calzada, Alejandro Ríos-Franceschi, Alessandro Catenazzi, Alessandro Ribeiro Morais, Alex Figueroa, Alexander Kupfer, Alex Rebelo, Alex Ttito, Alex Villegas, Alexander González, Alexander Shepack, Alexandre Pinheiro de Almeida, Alfonso Miranda Leiva, Alfredo Pedroso, Alfredo Salvador, Ali Qashqaei, Allen Allison, Álvaro Román, Amaël Borzée, Amanda Belén Quezada Riera, Amanda Haigh, Amatha Wickramasinghe, Amir Hamidy, Amit Hegde, An Martel, Ana Almendariz, Ana Longo, Anand Padhye, Anchalee Aowphol, Anderson Jean, Andolalao Rakotoarison, André Pansonato, Andrea Terán, Andreas Hertz, Andreas Kay, Andreas Schmitz, Andrés Aguayo, Andres Maletzky, Andrés Charrier, Andrés García Aguayo, Andrés Merino-Viteri, Andrés Camilo Montes-Correa, Andrés Posso-Terranova, Andrés Rymel Acosta-Galvis, Andrés Valenzuela, Andrew J. Crawford, Andrew Cunningham, Andrew Glusenkamp, Andrew Gray, Andrew Plumptre, Andrew Snyder, Andrew Turner, Andrew Watson, Andrew Whitworth, Angel Romero, Ángel Sosa-Bartuano, Angel Soto, Angelica Crottini, Annemarie Ohler, Annika Hillers, Ansel Fong G., Anslem de Silva, Antoine Fouquet, Antonín Krása, Antonio Cadiz, Antonio Muñoz-Alonso, Antonio Ramírez Velázquez, Antonio Romano, Anuar Shahrul, Argelina Blanco-Torres, Ariadne Angulo, Ariadne Fares Sabbag, Ariel Rodríguez, Arístides García Vinalay, Arlene Cardozo-Urdaneta, Arlo Hinckley, Artem A. Kidov, Arturo Muñoz Saravia, Arturo Salmeron, Arvin C. Diesmos, Atherton de Villiers, Atsushi Tominaga, Attapol Rujirawan, Audrey Owens, Austin Fitzgerald, Awadh Al Johany, Awal Riyanto, Axel Kwet, Aziz Avci, Balint Halpern, Barbod Safaei Mahroo, Barkha Subba, Barnagleison Silva Lisboa, Basundhara Chettri, Belisario Cepeda-Quilindo, Ben Evans, Ben Wielstra, Ben D. Bell, Benedikt Schmidt, Benjamin Tapley, Bhaskar Saikia, Bianca Berneck, Billie Harrison, Bin Wang, Biraj Shrestha, Blake Klocke, Bo Cai, Bo Wen, Boris Blotto, Boris Tuniyev, Branko Hilje, Breda Zimkus, Brian Crawford, Brian

Gratwicke, Brian Halstead, Brian Hudgens, Brian Kubicki, Bruce Christman, Bruce Means, Bruno Timbe-Borja, Bryan Stuart, Burhan Tjaturadi, Byron Wilson, Caio Marinho Mello, Caleb Ofori-Boateng, Camila Castro Carrasco, Carl Hutter, Carlos Camp, Carlos Marin, Carlos C. Martínez Rivera, Carlos Pacheco, Carlos Valle-Piñuela, Carlos R. Vásquez-Almazán, Carlos Frederico Duarte da Rocha, Carmen Úbeda, Carol Hughes, Carola A. Haas, Carolina Reyes-Puig, Caroline Zank, Caroline Batistim Oswald, Cathy Brown, Ceal Klingler, Célio Fernando Baptista Haddad, Celsa Señaris, César Aguilar Puntriano, César Barrio-Amorós, César Cuevas, César Jaramillo, César Malambo, Chandramouli S.R., Charif Tala, Chatoan Tesia, Chelmala Srinivasulu, Chou Wenhao, Chris Beirne, Chris Dahl, Chris Phipps, Chris Portway, Christian Supsup, Christine Strüssmann, Christophe Dufresnes, Christopher Austin, Christopher Evelyn, Christoph Grünwald, Christopher Norment, Christopher Raxworthy, Cinthia Aguirre Brasileiro, Claude Miaud, Claudia Fabiola Cortez Fernández, Claudia Corti, Claudia Koch, Claudia Molina, Claudia María Vélez, Claudio Correa, Claudio Azat, Conrad J. Hoskin, Corinne Richards-Zawacki, Cristian Marte, Cristiano Liuzzi, Cristiano Nogueira, Cristopher Antúnez, Cuong The Pham, Cybele Sabino Lisboa, Daiana Paola Ferraro, Daicus Belabut, Dale Roberts, Damany Calder, Damion L. Whyte, Dan Cogălniceanu, Daniel Jablonski, Daniel Ariano-Sánchez, Daniel Cassiano Lima, Daniel Chávez Jácome, Daniel Davila, Daniel Escoriza, Daniel Medina, Daniel Mejía-Vargas, Daniel Oliveira Mesquita, Daniel Padilla Jiménez, Daniel Portik, Daniel Rodríguez, Daniele Canestrelli, Daniele Salvi, Daniele Seglie, Danilo Balete, Danny Boiano, Dario Cardozo, Darrell Frost, David Beamer, David Blackburn, David Donaire-Barroso, David J. Gower, David Hillis, David Hunter, David McLeod, David Newell, David Steen, David Tarkhnishvili, David Wake, Dawne Emery, Deanna H. Olson, Déborah Praciano de Castro, Delia Basanta, Delio Baeta, Denis Vallan, Deon Gilbert, Devin Edmonds, Diana Székely, Diego Armijos-Ojeda, Diego Baldo, Diego Barrasso, Diego Ferrer, Diego Gómez, Diego Janisch Alvares, Diego Ortiz, Diego A. Flores Padron, Diego F. Cisneros-Heredia, Diego José Santana, Dinal Samarasinghe, Dinesh Gabadage, Ding-Qi Rao, Djoko Iskandar, Doade Yang, Donan Satria, Dushantha Kandambi, Eddie Rakotondrasoa, Edgar Bernal Castro, Edgar Jose, Edgar Lehr, Edgardo Flores, Edgardo Griffith, Edmund Leo Rico, Edmundo Perez Ramos, Edson Cortez, Eduardo Boza, Eduardo Pineda Arredondo, Eduardo Sanabria, Eduardo Schaefer, Eduardo J. Rodríguez-Rodríguez, Eduardo José dos Reis Dias, Edvárd Mizsei, Edward Aruna, Edward Camargo, Edwin Gómez-Méndez, Edwin Tambara, Edwin E. Infante-Rivero, El Hassan El Mouden, Elaine Maria Lucas Gonsales, Eli Geffen, Eli Greenbaum, Elizabeth Bell, Elizabeth Jockusch, Elizah Nagombi, Elnaz Najafi-Majd, Elodie Courtois, Elson Meneses-Pelayo, Emanuel Morán, Emanuel Teixeira da Silva, Emerson Sy, Enerit Sacbanaku, Enrico Lunghi, Enrique La Marca, Enrique Ramos, Enzo Isaak Carias Perdomo, Eric Van Den Berghe, Erick Arias, Erika Ximena Cruz-Rodríguez, Esteban Lavilla, Estefany Cano, Estefany Illueca, Evan S.H. Quah, Evan Twomey, Evy Arida, Eyup Başkale, Fabio Pupin, Fábio Hepp, Fábio Maffei, Fabio Leonardo Meza-Joya, Fang Yan, Fargang Torki, Fausto Nomura, Fausto Siavichay Pesántez, Federico Bolaños, Federico Kacoliris, Felipe Camurugi Almeida Guimarães, Felipe Rabanal, Felipe Sá Fortes Leite, Fernanda de Pinho Werneck, Fernando Bird Pico, Fernando Castro, Fernando Rojas-Runjaic, Fernando Vargas-Salinas, Fikirte Gebresenbet, Firoz Ahmed, Flavia Netto, Florina Stănescu, Francesca Protti, Francesco Ficetola, Francesco Lillo, Francisco Brusquetti, Francisco S. Álvarez, Franco Andreone, Francois Becker, Frank Glaw, Frank Pasmans, Franklin Enrique Castañeda, Fred Kraus, Gabriel Calapa, Gabriel Lobos, Gabriel Seneg, Gabriela Agostini, Gabriela Parra-Olea, Gail Ross, Gang Wei, Gary Bucciarelli, Gayathri Sreedharan, Geng Baorong, Geoffrey

Hammerson, Geoffrey Heard, George Lonsdale, Georgi Popgeorgiev, Georgina Santos-Barrera, Geraldo Jorge Barbosa de Moura, Gerardo Chaves, Germán Chavez, Gilbert Adum, Gilbert Alvarado Barboza, Gilles Pottier, Gilson Rivas, Gina DellaTogna, Giovanna Chipana, Giovanni Chaves Portilla, Giulia Tessa, Giussepe Gagliardi, Glib Mazepa, Gonçalo Rosa, Gopalakrishna Bhatta, Göran Nilson, Govindappa Venu, Graeme Gillespie, Graham Reynolds, Grant Webster, Greg Hollis, Gregor Aljančič, Guido F. Medina-Rangel, Guillermo Velo-Antón, Guinevere Wogan, Gustavo Casas, Gustavo Fermín, Gustavo Pisso, Gustavo Ruano-Fajardo, Gustavo González-Durán, H.T. Lalremsanga, Hana Putra Wicesa, Harald Hinkel, Harith Farooq, Harry Hines, Héctor Zumbado Ulate, Heidi Ross, Helen Díaz Páez, Helen Meredith, Hellen Kurniati, Hernán Pastore, Hidetoshi Ota, Hiva Faizi, Holly Siow, Ian Vogel, Ibere F. Machado, Idriss Bouam, Ignacio De la Riva, Igor Luis Kaefer, Ikuo Miura, Ilias Strachinis, Indraneil Das, Iñigo Martínez-Solano, Irina Maslova, Iris Holmes, Itamar Alves Martins, Iuri Ribeiro Dias, Iván Ahumada Carrillo, Ivan Nunes, Izabela Menezes Barata, J. Amanda Delgado C., Jaime Bosch, Jaime Culebras, Jaime Pefaur, Jaime Smith, Jaime Villacampa, James Aparicio, James Harvey, James Rorabaugh, James Randall McCranie, Jamie Voyles, Jan Pauel, Jana C. Riemann, Janak Khatiwada, Jason Brown, Javier García-Gutiérrez, Javier Sunyer, Javiera Cisternas, Jayaditya Purkayastha, Jayanta Kumar Roy, Jean-Marc Thirion, Jeanne Tarrant, Jeet Sukumaran, Jef Jaeger, Jeff Dawson, Jeff Humphries, Jeffrey Hall, Jelka Crnobrnja-Isailović, Jennifer Dever, Jennifer Jones, Jenny Loda, Jeremy Feinberg, Jeremy Klank, Jeremy Lindsell, Jeroen Speybroeck, Jesse Delia, Jessica Galvez, Jesús Manzanilla, Jiang Jianping, Jian-Huan Yang, Jichao Wang, Jigme Tshelthrim Wangyal, Jihène Ben Hassine, Jill Newman, Jim Lee, Jimmy Alexander Guerrero-Vargas, Jin-Long Ren, Jing Chai, Jing Che, João Filipe Riva Tonini, João Victor Andrade Lacerda, Jodi J.L. Rowley, Joe Robb, Johannes Els, Johannes Penner, John Cleghorn, John Lamoreux, John MacGregor, John Maerz, John Measey, John Murphy, John Palis, John Phillips, John Poynton, John Tupy, John Wilkinson, John O. Cossel, John Roger Downie, Joie de Leon, Jonah van Beijnen, Jonathan Campbell, Jonathan Kolby, Jong Sik Choe, Jonh Mueses-Cisneros, Jorge Brito, Jorge García, Jorge Guerrel, Jos Kielgast, José Langone, José Perez, José Vicente Rueda, José Francisco Cáceres Andrade, José Luis Aguilar-López, José Luis Vieira, José Manuel Padial, José Rances Caicedo Portilla, Jose Vincente Rueda-Almonacid, Joseph Mendelson, Joseph Pechmann, Joseph J. Apodaca, Josiah Townsend, Josimar Estrella, Josue Ramos Galdámez, Juan Abarca Alvarado, Juan Guayasamin, Juan Carlos Chaparro, Juan Carlos Cusi, Juan Carlos Ortiz, Juan Carlos Sánchez, Juan David Loaiza, Juan Emiro Carvajal Cogollo, Juan Fernando Webster Bernal, Juan Pablo González de la Vega, Juan Pablo Ramírez, Juan Pablo Reyes, Juan Ramón Fernández Cardenete, Judit Vörös, Julian Faivovich, Julián N. Lescano, Juliane Petry de Carli Monteiro, Julie Razafimanahaka, Justin Gerlach, K. P. Dinesh, K.P. Rajkumar, Kotambylu Vasudeva Gururaja, Kadaba Shamanna Seshadri, Kalamanji Govindaiah Girish, Kanishka Ukuwela, Kanto Nishikawa, Karina Núñez, Karoline Ceron, Karthikeyan Vasudevan, Katherine Krynak, Kathleen Webster, Katja Poboljsaj, Kaushik Deuti, Keerthi Hemkant, Keerthi Krutha, Kelly Irwin, Kelum Manamendra-Arachchi, Ken Wray, Kevin Carter, Kevin Hamed, Kevin Messenger, Khaled Merabet, Kim Howell, Kimberly Castro, Kimberly Stephenson, Kin Onn Chan, Konstantinos Sotiropoulos, Koshiro Eto, Krishna Komanduri, Kristiina Ovaska, Krushnamegh Kunte, Krystal Tolley, Laura Bravo, Laura Sandberger-Loua, Laura Cecilia Pereyra, Leandro Alves da Silva, Leandro João Carneiro de Lima Moraes, Lee Grismer, Lee Kats, Leomerth Lacruz, Leonardo Vignoli, Leonardo Moreira, Leslie Minter, Leticia Afuang, Li Cheng, Li Jia-tang, Liam Bolitho, Lindsey Thurman, Lingyun Xiao, Lior Blank, Lisa Paguntalan, Lisandro Morán, Lorena Quiroga, Louis du Preez, Luan

Thanh Nguyen, Luca Coppari, Lucas Barrientos, Lucas Ferrante, Lucas Batista Crivellari, Luciana Barreto Nascimento, Luciana Signorelli, Lucindo Gonzales, Luis Amador, Luis Canseco-Márquez, Luis Castillo Roque, Luis Coloma, Luis M. Díaz, Luis Elizondo, Luis Herrera, Luis Mamani Ccasa, Luis Zambrano, Luis Alberto Rueda-Solano, Luís Felipe Toledo, Luis Fernando Marin da Fonte, Luis Fernando Ribeiro, Luis Orlando Armesto Sanguino, Luiz Fernando Rocha Ugioni, Luke Easton, Madhava Botejue, Madhava Meegaskumbura, Madhushri Mudke, Mae Diesmos, Mael Dewynter, Maggie Haines, Manfredo Turcios Casco, Manuel Guayara, Manuel E. Acevedo, Manuel Morales, Manuel H. Bernal, Marc Hayes, Marcela Vidal, Marcelo Duarte Freire, Marcelo Felgueiras Napoli, Marcelo José Sturaro, Márcio Borges-Martins, Marco Méndez, Marco Rada, Marcos A. Ponce, Marcos Ramírez Zárate, Marcos Rodríguez, Marcos Vaira, Marcus Thadeu Teixeira Santos, Mareike Petersen, Maresa Scofield, Margarita Lampo, Maria Beatriz Pérez Lara, Maria Elena Cuello, Marina Rodes Blanco, Marinus Steven Hoogmoed, José Mario Solís Ramos, Mario H. Yánez-Muñoz, Marisol Pedregosa, Marius Burger, Mark Bailey, Mark D. Scherz, Mark Wilkinson, Mark-Oliver Rödel, Marta Bernardes, Marta Duré, Marvin Anganoy, Mary-Ruth Lowe, Masafumi Matsui, Matheus de Toledo Moroti, Mathieu Denoel, Matt Greenlees, Matthew Forrest, Matthew Niemiller, Matthew O'Donnell, Matthew Schlesinger, Matthieu Berroneau, Matthias Stoeck, Maura Santora, Mauricio Akmentins, Mauricio Ocampo, H. Mauricio Ortega-Andrade, Mauricio Pacheco, Mauricio Pareja, Mauricio Rivera-Correa, Max Dehling, Maxon Fildor, Mayke De Freitas, Md. Kamrul Hasan, M. Monirul H. Khan, Mendis Wickramasinghe, Mian Hou, Michael Barej, Michael Britton, Michael Cunningham, Michael Harvey, Michael Jowers, Michael Lannoo, Michael Lau, Michael Mahony, Michael McFadden, Michael Sisson, Michele Menegon, Michelle Abadie, Michelle Christman, Michelle C. Castellanos-Montero, Miguel Gómez Laporta, Miguel Landestoy, Miguel Urgilés, Miguel Vences, Mike Hudson, Mileidy Betancourth-Cundar, Min Mi-Sook, Mirco Solé, Mirna Garcia-Castillo, Mirza D. Kusrini, Misbahul Munir, Mistar Kamsi, Mizuki Takahashi, Moacir S. Tinôco, Mohammad Firoz Ahmed, Mohd. Abdul Muin, Mohini Mohan Borah, Moisés Escalona, Moises Kaplan, Mozafar Sharifi, Muhammad Rais, Murilo Sousa Andrade, N.A. Aravind, N'Goran G. Kouamé, Naalin Perera, Naitik Patel, Nancy Fairchild, Nate Engbrecht, Nathan Bendik, Natsuhiko Yoshikawa, Nayana Pradeep Kumara, Nayana Wijethilaka, Nazan Üzüm, Neelesh Dahanukar, Neftalí Ríos-López, Nereida Guerra Arevalo, Néstor Basso, Nethu Wickramasinghe, Nicholas Van Gilder, J. Nicolas Urbina-Cardona, Nikhil Dandekar, Nikhil Modak, Nikki Dyanne Realubit, Nikolay Poyarkov, Nina Bulakhova, Ninad Gosavi, Ninda Baptista, Nirhy Rabibisoa, Nono LeGrand Gonwouo, Norhayati Ahmad, Nurhayat Özdemir, Nzano Humtsoe, Octavio R. Rojas Soto, Oliver Hawlitschek, Oliver Quinteros, Olivier Guillaume, Olivier Pauwels, Omar Hernandez, Omar Rojas Padilla, Onil Ballestas, Orlando Ariel Garcés, Oscar Lasso-Alcalá, Oscar Jesús Damián-Baldeón, Oswaldo Cortés, Pablo Venegas, Parham Beyhaghi, Patricia Bejarano-Muñoz, Patricia A. Burrowes, Patricia Mendoza, Patrick Colombo, Patrick Malonza, Patrick McLaughlin, Patrick Ribeiro Sanches, Paul Granado, Paul Gutiérrez, Paul Hamilton, Paul Moler, Paul Oliver, Paul Székely, Paul Walker, Paul Y. Imbun, Paula Hanna Valdujo, Paulo Christiano de Anchietta Garcia, Pedro Galvis, Pedro Lopez del Castillo, Pedro Ivo Simões, Pedro Luiz Vieira Peloso, Perry Ong, Peter Heimes, Peter Trontelj, Petros Lymberakis, Phil Bishop, Philip Clerke, Philip de Pous, Philipp Wagner, Philippe Geniez, Philippe J.R. Kok, Pierre Razafindraibe, Pierre-André Crochet, Pierson Hill, Pipeng Li, Pradeep Samarawickrama, Pratyush Mohapatra, Priti Hebbar, Pritpal Soorae, Prudhvi Raj Gunturu, Rachel Montesinos Martins Pereira, Rachunliu Kamei, Rafael L. Joglar, Rafael Lajmanovich, Rafael Márquez,

Rafael Félix de Magalhães, Rafael Filgueira Jorge, Rainer Günther, Rainer Schulte, Rakshya Thapa, Ramachandran Kotharambath, Ramón Rivero, Randall Babb, Randall Jiménez Quirós, Raoul Manenti, Raphali Rodlis Andriantsimanarilafy, Raquel Betancourt, Raquel Hernández, Raul Maneyro, Rayna Bell, Rebecca Tarvin, Regina Medina, Reinaldo Aviles, Rémi Duguet, Renata Platenberg, Renato Morales, Renato Christensen Nali, Renato Neves Feio, Renee Catullo, Renoir J. Auguste, Reuber Albuquerque Brandão, Rhys Burns, Ricardo Cossio, Ricardo Miller, Ricardo Reques, Richard Highton, Richard Jenkins, Richard Kuyper, Richard Tinsley, Rick Lehtinen, Rita Cáceres Charneco, Rob Hopkins, Robert Fisher, Robert Hansen, Robert Murphy, Robert Powell, Roberto Alonso, Roberto Elías Piperis, Roberto Gutiérrez, Roberto Ibáñez, Roberto Luna Reyes, Robin Moore, Robin Suyesh, Robson Waldemar Ávila, Roby Nuñez, Rod Hitchmough, Rodrigo Aguayo, Rodrigo Cajade, Rodrigo Lingnau, Rodrigo Barbosa Ferreira, Rogério Pereira Bastos, Rohan Pethiyagoda, Roland Knapp, Ronald Crombie, Ronald Nussbaum, Ronald Zollinger, Ronaldo Lagat, Rosa Elena Zegarra, Ross MacCulloch, Ross Maynard, Roy Santa Cruz, Rubie Causaren, Rudolf von May, Rupert Mathwin, Rury Eprilurahman, Ruth Percino Daniel, S Harikrishnan, S. Blair Hedges, S.A.M Amer, S.P Vijayakumar, S.R. Ganesh, Sabitry Choudhury Bordoloi, Salim Busais, Sally Wren, Salomón Ramírez-Jaramillo, Salvador Carranza, Sam Cuenca, Samanta Iop, Sampath de Alwis Goonatilake, Sampath Udugampola, Samuel Turvey, Samuel Campos Gomides, Sandeep Das, Sandra Buckner, Sandra Diaz, Sandra Galeano, Sandra V. Flechas, Sandy Arroyo, Sanjay Molur, Sanoj Wijayasekara, Santiago Carreira, Santiago Castroviejo-Fisher, Santosh Bhattarai, Sara L. Ashpole, Sarah Mângia, Sarig Gafny, S.D. Biju, Scott Trageser, Scott Travers, Sean Graham, Sean Reilly, Sean Rovito, Sebastián Barrionuevo, Sebastián Lotzkat, Sebastian Kohn, Sergé Bogaerts, Sergio Rosset, Sergio Terán Juárez, Serkan Gül, Seyyed Saeed Hosseinian Yousefkhani, Shahrul Anuar, Shannon Behmke, Sharyn Marks, Sheila Pereira de Andrade, Sherif Baha El Din, Shingo Tanabe, Shu Chen, Silvia J. Robleto-Hernández, Simon Clulow, Simon P. Loader, Simon N. Stuart, Sixto J. Incháustegui, Skye Wassens, Sloane Jackson, Sofía Carvajal-Endara, Soumphthone Phimmachak, Sonali Garg, Sonam Phunstho, Sondra Vega, Soumia Fahd, Spartak Litvinchuk, Stanley Salazar, Stefan Lötters, Steffen Reichle, Stéphane Augros, Stephen Busack, Stephen Mahony, Stephen J. Richards, Steve Morey, Steven Anderson, Steven Gutiérrez, Steven Whitfield, Subarna Ghimire, Suélen da Silva Alves Saccol, Sumio Okada, Suranjan Karunarathna, Sushil Dutta, Sylvain Dubey, Tahar Slimani, Tainá Figueras Dorado Rodrigues, Tamara Osborne-Naikatini, Tanya Chan-Ard, Tao Thien Nguyen, Taran Grant, Tatianne P.F. Abreu-Jardim, Tatjana Dujsebayeva, Tatsuhiro Tokuda, Teddy Angarita Sierra, Tej Kumar Shrestha, Teresa Camacho Badani, Thaís Barreto Guedes da Costa, Thais Helena Condez, Thiago Silva-Soares, Thiago R. De Carvalho, Thomas Doherty-Bone, Thomas Ziegler, Thy Neang, Tiago Gomes do Santos, Tian Zhao, Tiffany Kosch, Timothy P. Cutajar, Tito Barros, Todd W. Pierson, Tom Brown, Tom Devitt, Tom Kirschey, Tom Martin, Tomohiko Shimada, Travis Taggart, Truong Q. Nguyen, Trupti D. Jadhav, Tutul Bortamuli, Twan Leenders, Ufuk Bülbül, Uğur Kaya, Ulmar Grafe, Ulrich Schulte, Umilaela Arifin, Uthpala Jayawardena, Vaclav Gvozdik, Valentina Caorsi, Valentina Posse, Valeria Corbalán, Valeria Franco, Valerija Zakšek, Vanda Lúcia Ferreira, Varad Bhagwan Giri, Victor Acosta Chaves, Victor Jiménez, Víctor Vargas, Victor Wasonga, Victor Zaracho, Victor G. Dill Orrico, Vincent Farallo, Vinicius Guerra Batista, Vishan Pushpamal, Vishnupriya Sankaraman, Vitor Carvalho-Rocha, Vladlen Henríquez, Walter Smith, Wan-Sheng Jiang, Wang Bin, Wang Jichao, Wang Jie, Waqas Ali, Wendy Bolaños, Wenhao Chou, Werner Conradie, Werther Pereira Ramalho, William Branch, William Flint, Wilmar Bolivar, Wouter Beukema, Xiaohong Chen, Xiaomao Zeng, Feng Xie,

Yang Daode, Yankho Chapeta, Yasuchika Misawa, Ying-Yong Wang, Yong Min Pui, Youszef Oliveira da Cunha Bitar, Yuan Zhigang, Yuechan Zhang, Yurii Kornilev, Yusnaviel García Padrón, Zaida Ortega Diago, Zeng Xiaomao, Zhao Wenge, Zhi-Tong Lyu, Zhiyong Yuan, Zhong Zhao, Mediyansyah, Mumpuni.

Supplementary Tables

<u>Supplementary Table 1</u>

Country	Total 1980–2004	Climate change effects	Dis-ease	Habitat loss/ degradation	Over-exploitation	Num-erous	Undet-ermined	Total 2004–2022	Climate change effects	Dis-ease	Habitat loss/ degradation	Over-exploitation	Num-erous	Undet-ermined	*Diff-erence*
Venezuela	**15**	0	14	1	0	0	0	**61**	50	1	10	0	0	0	**46**
Cameroon	**1**	0	0	0	1	0	0	**9**	0	9	0	0	0	0	**8**
Italy	**3**	0	0	3	0	0	0	**9**	0	8	0	0	0	1	**6**
United States	**10**	1	3	6	0	0	0	**16**	12	0	4	0	0	0	**6**
France	**1**	0	0	1	0	0	0	**6**	0	5	1	0	0	0	**5**
Guyana	**5**	0	0	5	0	0	0	**10**	2	0	8	0	0	0	**5**
Papua New Guinea	**3**	2	0	1	0	0	0	**8**	0	0	8	0	0	0	**5**
Japan	**1**	0	0	1	0	0	0	**5**	1	0	4	0	0	0	**4**
Seychelles	**0**	0	0	0	0	0	0	**4**	4	0	0	0	0	0	**4**
Nigeria	**1**	0	0	1	0	0	0	**4**	0	4	0	0	0	0	**3**
Türkyie	**1**	0	0	1	0	0	0	**4**	0	1	3	0	0	0	**3**
Austria	**0**	0	0	0	0	0	0	**2**	0	2	0	0	0	0	**2**
Bosnia and Herzegovina	**0**	0	0	0	0	0	0	**2**	0	2	0	0	0	0	**2**
Czechia	**0**	0	0	0	0	0	0	**2**	0	2	0	0	0	0	**2**
French Guiana	**1**	0	0	1	0	0	0	**3**	0	3	0	0	0	0	**2**
Germany	**0**	0	0	0	0	0	0	**2**	0	2	0	0	0	0	**2**
Ghana	**1**	0	0	1	0	0	0	**3**	0	0	3	0	0	0	**2**
Hungary	**0**	0	0	0	0	0	0	**2**	0	2	0	0	0	0	**2**
Korea, Republic of	**3**	0	0	3	0	0	0	**5**	0	0	5	0	0	0	**2**
Netherlands	**0**	0	0	0	0	0	0	**2**	0	2	0	0	0	0	**2**
Algeria	**0**	0	0	0	0	0	0	**1**	0	1	0	0	0	0	**1**
Andorra	**0**	0	0	0	0	0	0	**1**	0	1	0	0	0	0	**1**
Armenia	**0**	0	0	0	0	0	0	**1**	0	0	1	0	0	0	**1**
Belgium	**0**	0	0	0	0	0	0	**1**	0	1	0	0	0	0	**1**
Bulgaria	**0**	0	0	0	0	0	0	**1**	0	1	0	0	0	0	**1**
Canada	**0**	0	0	0	0	0	0	**1**	1	0	0	0	0	0	**1**
Cuba	**1**	0	1	0	0	0	0	**2**	1	1	0	0	0	0	**1**
Equatorial Guinea	**1**	0	0	0	1	0	0	**2**	0	2	0	0	0	0	**1**

**Country**	**Total 1980–2004**	Climate change effects	Dis-ease	Habitat loss/ degradation	Over-exploitation	Num-erous	Undet-ermined	**Total 2004–2022**	Climate change effects	Dis-ease	Habitat loss/ degradation	Over-exploitation	Num-erous	Undet-ermined	***Diff-erence***
Georgia	**0**	0	0	0	0	0	0	**1**	0	0	1	0	0	0	**1**
Greece	**0**	0	0	0	0	0	0	**1**	0	1	0	0	0	0	**1**
India	**0**	0	0	0	0	0	0	**1**	0	0	1	0	0	0	**1**
Liechtenstein	**0**	0	0	0	0	0	0	**1**	0	1	0	0	0	0	**1**
Luxembourg	**0**	0	0	0	0	0	0	**1**	0	1	0	0	0	0	**1**
Malaysia	**0**	0	0	0	0	0	0	**1**	0	0	1	0	0	0	**1**
North Macedonia	**0**	0	0	0	0	0	0	**1**	0	1	0	0	0	0	**1**
Poland	**0**	0	0	0	0	0	0	**1**	0	1	0	0	0	0	**1**
Portugal	**4**	0	0	4	0	0	0	**5**	0	3	2	0	0	0	**1**
Romania	**0**	0	0	0	0	0	0	**1**	0	1	0	0	0	0	**1**
San Marino	**0**	0	0	0	0	0	0	**1**	0	1	0	0	0	0	**1**
Serbia	**0**	0	0	0	0	0	0	**1**	0	1	0	0	0	0	**1**
Slovakia	**0**	0	0	0	0	0	0	**1**	0	1	0	0	0	0	**1**
Slovenia	**1**	0	0	1	0	0	0	**2**	0	2	0	0	0	0	**1**
Sri Lanka	**0**	0	0	0	0	0	0	**1**	0	0	0	0	1	0	**1**
Switzerland	**1**	0	0	1	0	0	0	**2**	0	2	0	0	0	0	**1**
Togo	**0**	0	0	0	0	0	0	**1**	0	0	1	0	0	0	**1**
Ukraine	**0**	0	0	0	0	0	0	**1**	0	1	0	0	0	0	**1**
United Kingdom	**0**	0	0	0	0	0	0	**1**	0	1	0	0	0	0	**1**
Albania	**1**	0	0	1	0	0	0	**1**	0	1	0	0	0	0	**0**
Croatia	**2**	0	0	2	0	0	0	**2**	0	2	0	0	0	0	**0**
Ethiopia	**1**	0	1	0	0	0	0	**1**	0	1	0	0	0	0	**0**
Gibraltar	**1**	0	0	1	0	0	0	**1**	0	0	1	0	0	0	**0**
Guinea	**1**	0	0	1	0	0	0	**1**	0	0	1	0	0	0	**0**
Martinique	**1**	0	1	0	0	0	0	**1**	1	0	0	0	0	0	**0**
Montenegro	**1**	0	0	1	0	0	0	**1**	0	1	0	0	0	0	**0**
Morocco	**1**	0	0	1	0	0	0	**1**	0	1	0	0	0	0	**0**
Nicaragua	**5**	0	4	1	0	0	0	**5**	3	0	2	0	0	0	**0**
Russian Federation	**1**	0	0	0	1	0	0	**1**	0	0	1	0	0	0	**0**
Spain	**6**	0	0	6	0	0	0	**6**	0	4	2	0	0	0	**0**
Cambodia	**1**	0	0	0	1	0	0	**0**	0	0	0	0	0	0	**-1**
Côte d'Ivoire	**2**	0	0	2	0	0	0	**1**	0	0	1	0	0	0	**-1**

Country	Total 1980–2004	Climate change effects	Dis-ease	Habitat loss/ degradation	Over-exploitation	Num-erous	Undet-ermined	Total 2004–2022	Climate change effects	Dis-ease	Habitat loss/ degradation	Over-exploitation	Num-erous	Undet-ermined	*Diff-erence*
Dominica	**1**	0	1	0	0	0	0	**0**	0	0	0	0	0	0	**-1**
Gabon	**1**	0	0	0	1	0	0	**0**	0	0	0	0	0	0	**-1**
Guadeloupe	**1**	0	1	0	0	0	0	**0**	0	0	0	0	0	0	**-1**
Hong Kong	**1**	0	0	0	1	0	0	**0**	0	0	0	0	0	0	**-1**
Indonesia	**1**	0	0	1	0	0	0	**0**	0	0	0	0	0	0	**-1**
Kyrgyzstan	**1**	0	0	1	0	0	0	**0**	0	0	0	0	0	0	**-1**
Lesotho	**1**	0	1	0	0	0	0	**0**	0	0	0	0	0	0	**-1**
Madagascar	**1**	0	0	1	0	0	0	**0**	0	0	0	0	0	0	**-1**
Montserrat	**1**	0	1	0	0	0	0	**0**	0	0	0	0	0	0	**-1**
Mozambique	**1**	0	0	1	0	0	0	**0**	0	0	0	0	0	0	**-1**
New Caledonia	**1**	0	1	0	0	0	0	**0**	0	0	0	0	0	0	**-1**
Paraguay	**1**	0	0	0	1	0	0	**0**	0	0	0	0	0	0	**-1**
Saint Kitts and Nevis	**1**	0	1	0	0	0	0	**0**	0	0	0	0	0	0	**-1**
Sao Tomé and Principe	**1**	0	0	1	0	0	0	**0**	0	0	0	0	0	0	**-1**
Tanzania	**2**	0	0	2	0	0	0	**1**	0	1	0	0	0	0	**-1**
Thailand	**1**	0	0	0	1	0	0	**0**	0	0	0	0	0	0	**-1**
Uruguay	**1**	0	0	1	0	0	0	**0**	0	0	0	0	0	0	**-1**
Vanuatu	**1**	0	1	0	0	0	0	**0**	0	0	0	0	0	0	**-1**
Virgin Islands, British	**1**	0	0	1	0	0	0	**0**	0	0	0	0	0	0	**-1**
Chile	**6**	0	1	3	2	0	0	**4**	2	1	1	0	0	0	**-2**
El Salvador	**2**	0	2	0	0	0	0	**0**	0	0	0	0	0	0	**-2**
Jamaica	**2**	0	2	0	0	0	0	**0**	0	0	0	0	0	0	**-2**
Kazakhstan	**2**	0	0	2	0	0	0	**0**	0	0	0	0	0	0	**-2**
Kenya	**2**	0	0	2	0	0	0	**0**	0	0	0	0	0	0	**-2**
Korea, Democratic People's Republic of	**3**	0	0	2	1	0	0	**1**	0	0	1	0	0	0	**-2**
Liberia	**2**	0	0	2	0	0	0	**0**	0	0	0	0	0	0	**-2**
New Zealand	**2**	0	2	0	0	0	0	**0**	0	0	0	0	0	0	**-2**
Virgin Islands, U.S.	**2**	0	1	1	0	0	0	**0**	0	0	0	0	0	0	**-2**
Zimbabwe	**2**	0	1	1	0	0	0	**0**	0	0	0	0	0	0	**-2**

Country	Total 1980–2004	Climate change effects	Dis-ease	Habitat loss/ degradation	Over-exploitation	Num-erous	Undet-ermined	Total 2004–2022	Climate change effects	Dis-ease	Habitat loss/ degradation	Over-exploitation	Num-erous	Undet-ermined	*Diff-erence*
Myanmar	**3**	0	0	0	3	0	0	**0**	0	0	0	0	0	0	**-3**
Philippines	**3**	0	0	1	2	0	0	**0**	0	0	0	0	0	0	**-3**
Viet Nam	**5**	0	0	0	5	0	0	**2**	0	0	1	1	0	0	**-3**
Belize	**4**	0	2	2	0	0	0	**0**	0	0	0	0	0	0	**-4**
Lao, PDR	**4**	0	0	0	4	0	0	**0**	0	0	0	0	0	0	**-4**
South Africa	**4**	0	1	3	0	0	0	**0**	0	0	0	0	0	0	**-4**
Argentina	**9**	0	5	3	1	0	0	**4**	2	1	1	0	0	0	**-5**
Dominican Republic	**6**	0	4	2	0	0	0	**0**	0	0	0	0	0	0	**-6**
Haiti	**6**	0	2	4	0	0	0	**0**	0	0	0	0	0	0	**-6**
Puerto Rico	**8**	1	6	1	0	0	0	**1**	1	0	0	0	0	0	**-7**
Bolivia	**26**	0	19	4	2	1	0	**16**	0	12	4	0	0	0	**-10**
Peru	**33**	0	21	9	1	2	0	**18**	1	7	10	0	0	0	**-15**
Australia	**29**	2	24	3	0	0	0	**13**	11	2	0	0	0	0	**-16**
Guatemala	**21**	0	13	8	0	0	0	**5**	2	0	3	0	0	0	**-16**
Panama	**34**	0	31	2	0	0	1	**18**	1	16	1	0	0	0	**-16**
China	**27**	0	0	4	23	0	0	**9**	0	0	5	4	0	0	**-18**
Honduras	**25**	0	20	5	0	0	0	**3**	0	0	3	0	0	0	**-22**
Brazil	**69**	0	28	41	0	0	0	**44**	27	2	15	0	0	0	**-25**
Mexico	**30**	0	22	8	0	0	0	**4**	0	1	3	0	0	0	**-26**
Costa Rica	**38**	0	35	1	0	0	2	**6**	3	2	1	0	0	0	**-32**
Ecuador	**81**	0	47	30	0	0	4	**37**	3	0	34	0	0	0	**-44**
Colombia	**74**	0	56	16	1	0	1	**22**	0	9	13	0	0	0	**-52**

Table 1. Number of species with status deteriorations per country in each time period. "Difference" column represents the difference in the number of species that deteriorated in status, comparing totals for 2004–2022 to 1980–2004 and ordered by largest increase to largest decrease.

Venezuela shows the largest increase in deteriorations since 2004 and is also the country with the most species that deteriorated in status during the 2004–2022 period. Most of these recent deteriorations are attributable to climate change effects (82%), whereas during 1980–2004 disease was recorded as the primary driver (93%) of category changes in the country.

Toward the end of the table are countries that have far fewer species that deteriorated in status in the 2004–2022 period compared to 1980–2004. The amphibian fauna of many countries – including Colombia, Ecuador, Costa Rica, Mexico, Honduras, Panama, Guatemala, Peru, Bolivia, Brazil, and Australia – were heavily impacted by disease in the earlier time period, but no longer appear to be experiencing declines at such a catastrophic scale.

Supplementary Table 2

ASG region	Years active	Countries or limits of the region
East Africa	2012–2016	Djibouti, South Sudan, Ethiopia, Somalia, Kenya, Uganda, Tanzania, Rwanda, Burundi, and eastern Democratic Republic of the Congo
West and Central Africa	2012–2020	Democratic Republic of Congo, Central African Republic, Republic of Congo, Gabon, Equatorial Guinea, Cameroon, Nigeria, Benin, Togo, Ghana, Burkina Faso, Côte d'Ivoire, Sierra Leone, Guinea, Guinea-Bissau, Gambia, Senegal, Liberia, Mali, Mauritania, southern Chad, and South Sudan
Mainland Southeast Asia	2013–2022	Cambodia, Lao People's Democratic Republic, Myanmar, Thailand, and Viet Nam
Madagascar	2014–2016	n/a
Panama	2014–2019	n/a
Mexico	2014–2020	n/a
Canada	2015	n/a
New Zealand	2015	n/a
Chile	2015–2018	n/a
Southern Africa	2016–2017	Angola, Zambia, Zimbabwe, Malawi, Mozambique, Botswana, Namibia, South Africa, Lesotho, and Eswatini
Melanesia	2016–2020	Maluku Islands, Papua, West Papua, Papua New Guinea, Solomon Islands, New Caledonia, Vanuatu, Palau, and Fiji
Peru	2016–2020	n/a
Colombia	2016–2021	n/a
Ecuador	2016–2021	n/a
Guiana Shield	2017–2018	Guyana, Suriname, French Guiana, northwestern Brazil, and southeastern Venezuela
Argentina	2017–2018	n/a
Indonesia	2017–2018	Sumatra, Java, Sulawesi, Kalimantan, the Sula Islands, and the Lesser Sunda Islands (east to Tanimbar, and including Timor-Leste)
Philippines	2017–2018	n/a
Malaysia and Singapore	2017–2018	Peninsular Malaysia, Malaysian Borneo (Sabah, Sarawak), Brunei Darussalam, Singapore
Caribbean	2017–2021	Anguilla, Antigua and Barbuda, Aruba, Bahamas, Barbados, Bermuda, Cayman Islands, Cuba, Dominica, Dominican Republic, Grenada, Guadeloupe, Haiti, Jamaica, Martinique, Montserrat, Netherlands Antilles, Puerto Rico, Saint Kitts and Nevis, Saint Lucia, Saint Vincent and the Grenadines, Trinidad and Tobago, Turks and Caicos Islands, and the Virgin Islands (British and U.S.)
Democratic People's Republic of Korea and Republic of Korea	2018–2022	n/a
Sri Lanka	2019–2020	n/a
Honduras	2019–2020	n/a
El Salvador	2019	n/a
Guatemala and Belize	2019–2020	n/a
Nicaragua	2019–2020	n/a
Costa Rica	2019–2020	n/a
Venezuela	2019–2020	n/a
Bolivia	2019–2020	n/a

China	2019–2021	n/a
Brazil	2019–2022	n/a
Europe	2019–2022	Åland Islands, Albania, Andorra, Austria, Belgium, Bosnia and Herzegovina, Bulgaria, Croatia, Czechia, Denmark, Estonia, Faroe Islands, Finland, France, Germany, Gibraltar, Greece, Greenland, Guernsey, Holy Sea (Vatican City State), Hungary, Iceland, Ireland, Isle of Man, Italy, Jersey, Latvia, Liechtenstein, Lithuania, Luxembourg, North Macedonia, Malta, Moldova, Monaco, Montenegro, Netherlands, Norway, Poland, Portugal, Romania, San Marino, Serbia, Slovakia, Slovenia, Spain, Svalbard and Jan Mayen, Sweden, Switzerland, Ukraine, and the United Kingdom
West and Central Asia	2019–2022	Armenia, Azerbaijan, Georgia, Kyrgyzstan, Tajikistan, Turkmenistan, and Uzbekistan
North Africa	2020	North Sudan, Northern Chad, northern Niger, northern Mali, northern Mauritania, Morocco, Algeria, Libya, Tunisia, and Egypt
Paraguay	2020	n/a
Uruguay	2020–2021	n/a
Commonwealth of Independent States and Mongolia	2020–2021	Russia, Mongolia, Kazakhstan, and Belarus
United States	2020–2021	n/a
Japan	2020–2021	n/a
Arabia and Middle East	2020–2022	Bahrain, Cyprus, Iran, Iraq, Israel, Jordan, Kuwait, Lebanon, Oman, Palestine, Qatar, Saudi Arabia, Syria, Türkyie, United Arab Emirates, and Yemen
Mainland South Asia	2020–2022	Afghanistan, Bangladesh, Bhutan, India, Nepal, and Pakistan
Australia	2020–2022	n/a

Table 2. IUCN SSC Amphibian Specialist Group regions. Includes a list of countries, where applicable, and the time period during which the region was actively updating Red List assessments.

Supplementary Table 3a

Order	Family	Genus and species	1980 backcasted Red List category	Primary driver of change between 1980–2004	2004 backcasted Red List category	2022 GAA2 Red List category
ANURA	ALLOPHRYNIDAE	*Allophryne relicta*	NT	Reduced-habitat	EN	EN
ANURA	ALYTIDAE	*Alytes dickhilleni*	NT	Reduced-habitat	VU	EN
ANURA	AROMOBATIDAE	*Allobates amissibilis*	LC	Reduced-habitat	VU	VU
ANURA	AROMOBATIDAE	*Allobates brunneus*	VU	Reduced-habitat	EN	EN
ANURA	AROMOBATIDAE	*Allobates mcdiarmidi*	NT	Disease	CR	CR(PE)
ANURA	AROMOBATIDAE	*Allobates ranoides*	CR	Disease	CR(PE)	CR(PE)
ANURA	AROMOBATIDAE	*Anomaloglossus kaiei*	LC	Reduced-habitat	NT	EN
ANURA	AROMOBATIDAE	*Anomaloglossus tepequem*	VU	Reduced-habitat	CR(PE)	CR(PE)
ANURA	AROMOBATIDAE	*Aromobates alboguttatus*	EN	Disease	CR(PE)	CR(PE)
ANURA	AROMOBATIDAE	*Aromobates haydeeae*	EN	Disease	CR(PE)	CR(PE)
ANURA	AROMOBATIDAE	*Aromobates leopardalis*	VU	Disease	CR(PE)	CR(PE)
ANURA	AROMOBATIDAE	*Aromobates nocturnus*	VU	Disease	CR(PE)	CR(PE)
ANURA	AROMOBATIDAE	*Aromobates serranus*	EN	Disease	CR(PE)	CR(PE)
ANURA	ARTHROLEPTIDAE	*Leptopelis occidentalis*	LC	Reduced-habitat	NT	NT
ANURA	BRACHYCEPHALIDAE	*Brachycephalus actaeus*	LC	Reduced-habitat	EN	EN
ANURA	BRACHYCEPHALIDAE	*Brachycephalus sulfuratus*	LC	Reduced-habitat	NT	NT
ANURA	BRACHYCEPHALIDAE	*Brachycephalus vertebralis*	LC	Reduced-habitat	EN	EN
ANURA	BRACHYCEPHALIDAE	*Ischnocnema epipeda*	CR	Disease	CR(PE)	CR(PE)
ANURA	BUFONIDAE	*Anaxyrus baxteri*	CR	Disease	EW	EW
ANURA	BUFONIDAE	*Anaxyrus californicus*	NT	Reduced-habitat	EN	EN
ANURA	BUFONIDAE	*Anaxyrus canorus*	VU	Disease	EN	VU
ANURA	BUFONIDAE	*Anaxyrus houstonensis*	VU	Reduced-habitat	CR	CR
ANURA	BUFONIDAE	*Atelopus angelito*	EN	Disease	CR(PE)	CR(PE)
ANURA	BUFONIDAE	*Atelopus ardila*	EN	Disease	CR(PE)	CR(PE)
ANURA	BUFONIDAE	*Atelopus arthuri*	EN	Disease	CR(PE)	CR(PE)
ANURA	BUFONIDAE	*Atelopus balios*	EN	Disease	CR	CR
ANURA	BUFONIDAE	*Atelopus bomolochos*	EN	Disease	CR	CR
ANURA	BUFONIDAE	*Atelopus boulengeri*	CR	Disease	CR(PE)	CR(PE)
ANURA	BUFONIDAE	*Atelopus carbonerensis*	EN	Disease	CR(PE)	CR(PE)
ANURA	BUFONIDAE	*Atelopus chiriquiensis*	EN	Disease	CR(PE)	EX
ANURA	BUFONIDAE	*Atelopus chocoensis*	EN	Disease	CR(PE)	CR(PE)
ANURA	BUFONIDAE	*Atelopus chrysocorallus*	EN	Disease	CR	CR
ANURA	BUFONIDAE	*Atelopus coynei*	EN	Disease	CR	CR
ANURA	BUFONIDAE	*Atelopus cruciger*	NT	Disease	CR	CR

Order	Family	Genus and species	1980 backcasted Red List category	Primary driver of change between 1980–2004	2004 backcasted Red List category	2022 GAA2 Red List category
ANURA	BUFONIDAE	*Atelopus ebenoides*	EN	Disease	CR	CR(PE)
ANURA	BUFONIDAE	*Atelopus elegans*	VU	Disease	CR	EN
ANURA	BUFONIDAE	*Atelopus epikeisthos*	EN	Reduced-habitat	CR	CR
ANURA	BUFONIDAE	*Atelopus erythropus*	LC	Disease	CR	CR(PE)
ANURA	BUFONIDAE	*Atelopus eusebianus*	EN	Disease	CR	CR(PE)
ANURA	BUFONIDAE	*Atelopus eusebiodiazi*	VU	Disease	CR(PE)	CR(PE)
ANURA	BUFONIDAE	*Atelopus exiguus*	EN	Disease	CR	EN
ANURA	BUFONIDAE	*Atelopus famelicus*	EN	Disease	CR	CR
ANURA	BUFONIDAE	*Atelopus gigas*	CR	Disease	CR(PE)	CR(PE)
ANURA	BUFONIDAE	*Atelopus guanujo*	EN	Disease	CR(PE)	CR(PE)
ANURA	BUFONIDAE	*Atelopus halihelos*	LC	Disease	CR(PE)	CR(PE)
ANURA	BUFONIDAE	*Atelopus ignescens*	VU	Disease	CR	CR
ANURA	BUFONIDAE	*Atelopus longirostris*	EN	Disease	CR	CR
ANURA	BUFONIDAE	*Atelopus lozanoi*	EN	Disease	CR	CR
ANURA	BUFONIDAE	*Atelopus lynchi*	CR	Disease	CR(PE)	CR(PE)
ANURA	BUFONIDAE	*Atelopus manauensis*	LC	Reduced-habitat	EN	EN
ANURA	BUFONIDAE	*Atelopus marinkellei*	EN	Disease	CR	CR
ANURA	BUFONIDAE	*Atelopus mindoensis*	EN	Disease	CR	CR
ANURA	BUFONIDAE	*Atelopus minutulus*	CR	Disease	CR(PE)	CR(PE)
ANURA	BUFONIDAE	*Atelopus monohernandezii*	CR	Disease	CR(PE)	CR(PE)
ANURA	BUFONIDAE	*Atelopus mucubajiensis*	VU	Disease	CR	CR
ANURA	BUFONIDAE	*Atelopus muisca*	VU	Disease	CR	CR
ANURA	BUFONIDAE	*Atelopus nanay*	VU	Disease	CR	CR
ANURA	BUFONIDAE	*Atelopus nicefori*	CR	Disease	CR(PE)	CR(PE)
ANURA	BUFONIDAE	*Atelopus onorei*	CR	Disease	CR(PE)	CR(PE)
ANURA	BUFONIDAE	*Atelopus orcesi*	CR	Disease	CR(PE)	CR(PE)
ANURA	BUFONIDAE	*Atelopus oxyrhynchus*	EN	Disease	CR(PE)	CR(PE)
ANURA	BUFONIDAE	*Atelopus pachydermus*	VU	Disease	CR(PE)	CR(PE)
ANURA	BUFONIDAE	*Atelopus pastuso*	EN	Disease	CR(PE)	CR(PE)
ANURA	BUFONIDAE	*Atelopus patazensis*	LC	Disease	CR	CR
ANURA	BUFONIDAE	*Atelopus peruensis*	NT	Disease	CR	CR(PE)
ANURA	BUFONIDAE	*Atelopus petersi*	EN	Disease	CR(PE)	CR(PE)
ANURA	BUFONIDAE	*Atelopus petriruizi*	EN	Disease	CR(PE)	CR(PE)
ANURA	BUFONIDAE	*Atelopus pictiventris*	VU	Disease	CR(PE)	CR(PE)
ANURA	BUFONIDAE	*Atelopus pinangoi*	EN	Disease	CR	CR(PE)
ANURA	BUFONIDAE	*Atelopus planispina*	CR	Disease	CR(PE)	CR(PE)

Order	Family	Genus and species	1980 backcasted Red List category	Primary driver of change between 1980–2004	2004 backcasted Red List category	2022 GAA2 Red List category
ANURA	BUFONIDAE	*Atelopus podocarpus*	EN	Disease	CR(PE)	CR(PE)
ANURA	BUFONIDAE	*Atelopus pulcher*	VU	Disease	CR	VU
ANURA	BUFONIDAE	*Atelopus quimbaya*	EN	Disease	CR(PE)	CR(PE)
ANURA	BUFONIDAE	*Atelopus senex*	EN	Disease	EX	EX
ANURA	BUFONIDAE	*Atelopus sernai*	EN	Disease	CR(PE)	CR(PE)
ANURA	BUFONIDAE	*Atelopus simulatus*	VU	Disease	CR	CR(PE)
ANURA	BUFONIDAE	*Atelopus sonsonensis*	EN	Disease	CR(PE)	CR(PE)
ANURA	BUFONIDAE	*Atelopus sorianoi*	EN	Disease	CR(PE)	CR(PE)
ANURA	BUFONIDAE	*Atelopus spurrelli*	LC	Disease	VU	VU
ANURA	BUFONIDAE	*Atelopus subornatus*	EN	Disease	CR(PE)	CR(PE)
ANURA	BUFONIDAE	*Atelopus tamaense*	VU	Disease	CR	CR
ANURA	BUFONIDAE	*Atelopus tricolor*	LC	Disease	EN	CR
ANURA	BUFONIDAE	*Atelopus varius*	LC	Disease	CR	CR
ANURA	BUFONIDAE	*Atelopus zeteki*	EN	Disease	CR	CR(PEW)
ANURA	BUFONIDAE	*Capensibufo rosei*	EN	Reduced-habitat	CR	CR
ANURA	BUFONIDAE	*Dendrophryniscus davori*	LC	Reduced-habitat	CR	CR
ANURA	BUFONIDAE	*Dendrophryniscus lauroi*	LC	Reduced-habitat	EN	EN
ANURA	BUFONIDAE	*Incilius fastidiosus*	EN	Disease	CR(PE)	CR(PE)
ANURA	BUFONIDAE	*Incilius holdridgei*	VU	Disease	CR	CR
ANURA	BUFONIDAE	*Incilius majordomus*	CR	Disease	CR(PE)	CR(PE)
ANURA	BUFONIDAE	*Incilius periglenes*	LC	Disease	EX	EX
ANURA	BUFONIDAE	*Leptophryne cruentata*	EN	Reduced-habitat	CR	CR
ANURA	BUFONIDAE	*Melanophryniscus milanoi*	LC	Reduced-habitat	EN	EN
ANURA	BUFONIDAE	*Melanophryniscus montevidensis*	LC	Reduced-habitat	NT	NT
ANURA	BUFONIDAE	*Melanophryniscus moreirae*	LC	Reduced-habitat	NT	NT
ANURA	BUFONIDAE	*Melanophryniscus peritus*	CR	Disease	CR(PE)	CR(PE)
ANURA	BUFONIDAE	*Nannophryne cophotis*	NT	Disease	CR	CR(PE)
ANURA	BUFONIDAE	*Nectophrynoides asperginis*	VU	Reduced-habitat	CR	EW
ANURA	BUFONIDAE	*Nectophrynoides poyntoni*	CR	Reduced-habitat	CR(PE)	CR(PE)
ANURA	BUFONIDAE	*Nimbaphrynoides occidentalis*	VU	Reduced-habitat	CR	CR
ANURA	BUFONIDAE	*Peltophryne fluviatica*	CR	Reduced-habitat	CR(PE)	CR(PE)
ANURA	BUFONIDAE	*Peltophryne lemur*	EN	Reduced-habitat	CR	EN
ANURA	BUFONIDAE	*Rhaebo blombergi*	NT	Reduced-habitat	VU	CR
ANURA	BUFONIDAE	*Rhaebo caeruleostictus*	LC	Unknown	CR(PE)	CR(PE)
ANURA	BUFONIDAE	*Rhinella achalensis*	VU	Disease	EN	EN
ANURA	BUFONIDAE	*Rhinella chrysophora*	EN	Disease	CR(PE)	CR(PE)

Order	Family	Genus and species	1980 backcasted Red List category	Primary driver of change between 1980–2004	2004 backcasted Red List category	2022 GAA2 Red List category
ANURA	BUFONIDAE	*Rhinella leptoscelis*	LC	Disease	VU	NT
ANURA	BUFONIDAE	*Rhinella rubropunctata*	NT	Reduced-habitat	VU	VU
ANURA	BUFONIDAE	*Vandijkophrynus amatolicus*	EN	Reduced-habitat	CR	CR
ANURA	BUFONIDAE	*Vandijkophrynus inyangae*	LC	Disease	VU	VU
ANURA	CALYPTOCEPHALELLIDAE	*Calyptocephalella gayi*	LC	Over-exploited	VU	VU
ANURA	CENTROLENIDAE	*Centrolene ballux*	EN	Unknown	CR	EN
ANURA	CENTROLENIDAE	*Centrolene buckleyi*	LC	Reduced-habitat	VU	CR
ANURA	CENTROLENIDAE	*Centrolene condor*	LC	Reduced-habitat	NT	EN
ANURA	CENTROLENIDAE	*Centrolene geckoidea*	LC	Disease	CR(PE)	CR(PE)
ANURA	CENTROLENIDAE	*Centrolene medemi*	NT	Disease	CR	EN
ANURA	CENTROLENIDAE	*Centrolene pipilata*	CR	Disease	CR(PE)	CR(PE)
ANURA	CENTROLENIDAE	*Nymphargus armatus*	VU	Disease	CR	CR
ANURA	CENTROLENIDAE	*Nymphargus balionotus*	NT	Reduced-habitat	EN	EN
ANURA	CENTROLENIDAE	*Nymphargus bejaranoi*	LC	Disease	VU	EN
ANURA	CENTROLENIDAE	*Nymphargus colomai*	LC	Reduced-habitat	NT	EN
ANURA	CENTROLENIDAE	*Nymphargus lindae*	LC	Reduced-habitat	NT	EN
ANURA	CENTROLENIDAE	*Nymphargus megacheirus*	CR	Reduced-habitat	CR(PE)	CR(PE)
ANURA	CENTROLENIDAE	*Nymphargus truebae*	LC	Disease	CR	CR(PE)
ANURA	CENTROLENIDAE	*Rulyrana spiculata*	LC	Disease	VU	NT
ANURA	CENTROLENIDAE	*Vitreorana parvula*	LC	Reduced-habitat	VU	VU
ANURA	CERATOBATRACHIDAE	*Cornufer citrinospilus*	LC	Climate change effects	VU	VU
ANURA	CERATOPHRYIDAE	*Ceratophrys stolzmanni*	NT	Reduced-habitat	VU	VU
ANURA	CONRAUIDAE	*Conraua goliath*	NT	Over-exploited	EN	EN
ANURA	CRAUGASTORIDAE	*Craugastor adamastus*	CR	Reduced-habitat	CR(PE)	CR(PE)
ANURA	CRAUGASTORIDAE	*Craugastor anciano*	CR	Disease	EX	EX
ANURA	CRAUGASTORIDAE	*Craugastor andi*	VU	Disease	CR(PE)	CR(PE)
ANURA	CRAUGASTORIDAE	*Craugastor angelicus*	EN	Disease	CR	CR
ANURA	CRAUGASTORIDAE	*Craugastor catalinae*	EN	Disease	CR(PE)	CR(PE)
ANURA	CRAUGASTORIDAE	*Craugastor chrysozetetes*	CR	Reduced-habitat	CR(PE)	CR(PE)
ANURA	CRAUGASTORIDAE	*Craugastor coffeus*	EN	Disease	CR	CR
ANURA	CRAUGASTORIDAE	*Craugastor cruzi*	CR	Disease	CR(PE)	CR(PE)
ANURA	CRAUGASTORIDAE	*Craugastor emcelae*	EN	Disease	CR	CR
ANURA	CRAUGASTORIDAE	*Craugastor emleni*	EN	Disease	CR	EN
ANURA	CRAUGASTORIDAE	*Craugastor epochthidius*	EN	Disease	CR(PE)	CR(PE)
ANURA	CRAUGASTORIDAE	*Craugastor escoces*	EN	Disease	CR	CR
ANURA	CRAUGASTORIDAE	*Craugastor fecundus*	EN	Disease	CR(PE)	CR(PE)

Order	Family	Genus and species	1980 backcasted Red List category	Primary driver of change between 1980–2004	2004 backcasted Red List category	2022 GAA2 Red List category
ANURA	CRAUGASTORIDAE	*Craugastor fleischmanni*	EN	Disease	CR	CR
ANURA	CRAUGASTORIDAE	*Craugastor greggi*	EN	Disease	CR	EN
ANURA	CRAUGASTORIDAE	*Craugastor gulosus*	EN	Disease	CR	CR
ANURA	CRAUGASTORIDAE	*Craugastor laevissimus*	LC	Disease	EN	EN
ANURA	CRAUGASTORIDAE	*Craugastor merendonensis*	VU	Disease	CR(PE)	CR(PE)
ANURA	CRAUGASTORIDAE	*Craugastor milesi*	EN	Disease	CR	CR
ANURA	CRAUGASTORIDAE	*Craugastor myllomyllon*	CR	Reduced-habitat	CR(PE)	EX
ANURA	CRAUGASTORIDAE	*Craugastor obesus*	EN	Disease	CR	CR
ANURA	CRAUGASTORIDAE	*Craugastor olanchano*	EN	Disease	CR(PE)	CR(PE)
ANURA	CRAUGASTORIDAE	*Craugastor omoaensis*	CR	Disease	EX	EX
ANURA	CRAUGASTORIDAE	*Craugastor phasma*	VU	Disease	CR(PE)	CR(PE)
ANURA	CRAUGASTORIDAE	*Craugastor ranoides*	LC	Disease	CR	CR
ANURA	CRAUGASTORIDAE	*Craugastor rhyacobatrachus*	EN	Disease	CR(PE)	CR(PE)
ANURA	CRAUGASTORIDAE	*Craugastor sabrinus*	NT	Reduced-habitat	EN	NT
ANURA	CRAUGASTORIDAE	*Craugastor saltuarius*	EN	Disease	CR(PE)	CR(PE)
ANURA	CRAUGASTORIDAE	*Craugastor sandersoni*	VU	Disease	EN	EN
ANURA	CRAUGASTORIDAE	*Craugastor stadelmani*	EN	Disease	CR	CR
ANURA	CRAUGASTORIDAE	*Craugastor tabasarae*	EN	Disease	CR	CR
ANURA	CRAUGASTORIDAE	*Craugastor taurus*	VU	Disease	CR	EN
ANURA	CRAUGASTORIDAE	*Craugastor trachydermus*	CR	Reduced-habitat	CR(PE)	CR(PE)
ANURA	CRAUGASTORIDAE	*Euparkerella robusta*	EN	Reduced-habitat	CR	CR
ANURA	CRAUGASTORIDAE	*Holoaden bradei*	CR	Disease	CR(PE)	CR(PE)
ANURA	CRAUGASTORIDAE	*Lynchius simmonsi*	LC	Reduced-habitat	NT	EN
ANURA	CRAUGASTORIDAE	*Microkayla guillei*	VU	Reduced-habitat	CR	CR
ANURA	CRAUGASTORIDAE	*Noblella lochites*	LC	Reduced-habitat	NT	EN
ANURA	CRAUGASTORIDAE	*Oreobates remotus*	LC	Reduced-habitat	EN	EN
ANURA	CRAUGASTORIDAE	*Pristimantis albericoi*	CR	Reduced-habitat	CR(PE)	CR(PE)
ANURA	CRAUGASTORIDAE	*Pristimantis anotis*	CR	Disease	CR(PE)	CR(PE)
ANURA	CRAUGASTORIDAE	*Pristimantis barrigai*	LC	Reduced-habitat	VU	CR
ANURA	CRAUGASTORIDAE	*Pristimantis bernali*	CR	Disease	CR(PE)	CR(PE)
ANURA	CRAUGASTORIDAE	*Pristimantis cacao*	EN	Disease	CR	CR
ANURA	CRAUGASTORIDAE	*Pristimantis caprifer*	VU	Disease	CR	CR
ANURA	CRAUGASTORIDAE	*Pristimantis chrysops*	EN	Disease	CR	CR
ANURA	CRAUGASTORIDAE	*Pristimantis cosnipatae*	EN	Disease	CR	CR
ANURA	CRAUGASTORIDAE	*Pristimantis deinops*	EN	Disease	CR	CR
ANURA	CRAUGASTORIDAE	*Pristimantis duellmani*	VU	Disease	CR	CR

Order	Family	Genus and species	1980 backcasted Red List category	Primary driver of change between 1980–2004	2004 backcasted Red List category	2022 GAA2 Red List category
ANURA	CRAUGASTORIDAE	*Pristimantis hamiotae*	CR	Reduced-habitat	CR(PE)	CR(PE)
ANURA	CRAUGASTORIDAE	*Pristimantis minimus*	LC	Reduced-habitat	NT	EN
ANURA	CRAUGASTORIDAE	*Pristimantis molybrignus*	VU	Disease	CR(PE)	CR(PE)
ANURA	CRAUGASTORIDAE	*Pristimantis nangaritza*	LC	Reduced-habitat	VU	CR
ANURA	CRAUGASTORIDAE	*Pristimantis paquishae*	LC	Reduced-habitat	VU	CR
ANURA	CRAUGASTORIDAE	*Pristimantis parvillus*	LC	Reduced-habitat	NT	VU
ANURA	CRAUGASTORIDAE	*Pristimantis phragmipleuron*	CR	Reduced-habitat	CR(PE)	CR(PE)
ANURA	CRAUGASTORIDAE	*Pristimantis pugnax*	NT	Disease	CR	CR
ANURA	CRAUGASTORIDAE	*Pristimantis signifer*	EN	Disease	CR	CR
ANURA	CRAUGASTORIDAE	*Pristimantis silverstonei*	VU	Disease	EN	VU
ANURA	CRAUGASTORIDAE	*Pristimantis yantzaza*	LC	Reduced-habitat	NT	EN
ANURA	CRAUGASTORIDAE	*Strabomantis anomalus*	NT	Reduced-habitat	CR	CR
ANURA	CRAUGASTORIDAE	*Strabomantis bufoniformis*	LC	Disease	VU	EN
ANURA	CRAUGASTORIDAE	*Strabomantis cadenai*	CR	Reduced-habitat	CR(PE)	CR(PE)
ANURA	CRAUGASTORIDAE	*Strabomantis cerastes*	LC	Reduced-habitat	CR	CR
ANURA	CRAUGASTORIDAE	*Strabomantis cheiroplethus*	VU	Disease	EN	EN
ANURA	CRAUGASTORIDAE	*Strabomantis cornutus*	LC	Disease	EN	EN
ANURA	CRAUGASTORIDAE	*Strabomantis necerus*	VU	Disease	CR(PE)	CR(PE)
ANURA	CRAUGASTORIDAE	*Yunganastes ashkapara*	LC	Reduced-habitat	EN	EN
ANURA	CRAUGASTORIDAE	*Yunganastes fraudator*	LC	Disease	NT	VU
ANURA	CRAUGASTORIDAE	*Yunganastes pluvicanorus*	LC	Disease	NT	VU
ANURA	CYCLORAMPHIDAE	*Cycloramphus catarinensis*	CR	Disease	CR(PE)	CR(PE)
ANURA	CYCLORAMPHIDAE	*Cycloramphus cedrensis*	CR	Disease	CR(PE)	CR(PE)
ANURA	CYCLORAMPHIDAE	*Cycloramphus diringshofeni*	CR	Disease	CR(PE)	CR(PE)
ANURA	CYCLORAMPHIDAE	*Cycloramphus granulosus*	CR	Disease	CR(PE)	CR(PE)
ANURA	CYCLORAMPHIDAE	*Cycloramphus izecksohni*	LC	Reduced-habitat	EN	EN
ANURA	CYCLORAMPHIDAE	*Cycloramphus ohausi*	CR	Disease	CR(PE)	CR(PE)
ANURA	CYCLORAMPHIDAE	*Cycloramphus semipalmatus*	CR	Disease	CR(PE)	CR(PE)
ANURA	CYCLORAMPHIDAE	*Cycloramphus stejnegeri*	CR	Disease	CR(PE)	CR(PE)
ANURA	CYCLORAMPHIDAE	*Cycloramphus valae*	CR	Disease	CR(PE)	CR(PE)
ANURA	CYCLORAMPHIDAE	*Thoropa lutzi*	CR	Disease	CR(PE)	CR(PE)
ANURA	CYCLORAMPHIDAE	*Thoropa petropolitana*	CR	Disease	CR(PE)	CR(PE)
ANURA	DENDROBATIDAE	*Andinobates viridis*	EN	Disease	CR	CR(PE)
ANURA	DENDROBATIDAE	*Ectopoglossus atopoglossus*	CR	Disease	CR(PE)	CR(PE)
ANURA	DENDROBATIDAE	*Hyloxalus abditaurantius*	NT	Disease	CR(PE)	CR(PE)
ANURA	DENDROBATIDAE	*Hyloxalus anthracinus*	VU	Disease	CR	CR

Order	Family	Genus and species	1980 backcasted Red List category	Primary driver of change between 1980–2004	2004 backcasted Red List category	2022 GAA2 Red List category
ANURA	DENDROBATIDAE	*Hyloxalus delatorreae*	EN	Disease	CR	CR
ANURA	DENDROBATIDAE	*Hyloxalus edwardsi*	CR	Reduced-habitat	CR(PE)	CR(PE)
ANURA	DENDROBATIDAE	*Hyloxalus elachyhistus*	LC	Disease	VU	LC
ANURA	DENDROBATIDAE	*Hyloxalus exasperatus*	EN	Unknown	CR(PE)	CR(PE)
ANURA	DENDROBATIDAE	*Hyloxalus fallax*	CR	Unknown	CR(PE)	CR(PE)
ANURA	DENDROBATIDAE	*Hyloxalus fascianigrus*	VU	Disease	CR	VU
ANURA	DENDROBATIDAE	*Hyloxalus infraguttatus*	LC	Reduced-habitat	NT	VU
ANURA	DENDROBATIDAE	*Hyloxalus lehmanni*	LC	Disease	NT	NT
ANURA	DENDROBATIDAE	*Hyloxalus maquipucuna*	CR	Disease	CR(PE)	CR(PE)
ANURA	DENDROBATIDAE	*Hyloxalus marmoreoventris*	CR	Disease	CR(PE)	CR(PE)
ANURA	DENDROBATIDAE	*Hyloxalus mystax*	LC	Reduced-habitat	NT	EN
ANURA	DENDROBATIDAE	*Hyloxalus peculiaris*	VU	Reduced-habitat	CR(PE)	CR(PE)
ANURA	DENDROBATIDAE	*Hyloxalus pulchellus*	NT	Disease	VU	NT
ANURA	DENDROBATIDAE	*Hyloxalus pumilus*	CR	Disease	CR(PE)	CR(PE)
ANURA	DENDROBATIDAE	*Hyloxalus ruizi*	CR	Reduced-habitat	CR(PE)	CR(PE)
ANURA	DENDROBATIDAE	*Hyloxalus shuar*	LC	Disease	CR	CR
ANURA	DENDROBATIDAE	*Hyloxalus vertebralis*	VU	Disease	CR	VU
ANURA	DENDROBATIDAE	*Minyobates steyermarki*	VU	Reduced-habitat	CR	CR
ANURA	DENDROBATIDAE	*Oophaga arborea*	EN	Disease	CR	CR
ANURA	DENDROBATIDAE	*Oophaga lehmanni*	EN	Over-exploited	CR	CR
ANURA	DENDROBATIDAE	*Oophaga speciosa*	EN	Disease	EX	EX
ANURA	DENDROBATIDAE	*Oophaga sylvatica*	LC	Reduced-habitat	NT	NT
ANURA	DENDROBATIDAE	*Paruwrobates andinus*	CR	Disease	CR(PE)	CR(PE)
ANURA	DENDROBATIDAE	*Silverstoneia nubicola*	LC	Disease	NT	VU
ANURA	DICROGLOSSIDAE	*Limnonectes macrocephalus*	LC	Reduced-habitat	NT	NT
ANURA	DICROGLOSSIDAE	*Limnonectes magnus*	LC	Over-exploited	NT	NT
ANURA	DICROGLOSSIDAE	*Limnonectes namiyei*	NT	Reduced-habitat	EN	EN
ANURA	DICROGLOSSIDAE	*Limnonectes visayanus*	LC	Over-exploited	NT	NT
ANURA	DICROGLOSSIDAE	*Nanorana quadranus*	LC	Over-exploited	NT	NT
ANURA	DICROGLOSSIDAE	*Nanorana sichuanensis*	LC	Over-exploited	VU	VU
ANURA	DICROGLOSSIDAE	*Nanorana unculuanus*	NT	Over-exploited	EN	VU
ANURA	DICROGLOSSIDAE	*Nanorana yunnanensis*	LC	Over-exploited	VU	VU
ANURA	DICROGLOSSIDAE	*Quasipaa boulengeri*	LC	Over-exploited	VU	VU
ANURA	DICROGLOSSIDAE	*Quasipaa jiulongensis*	NT	Over-exploited	VU	VU
ANURA	DICROGLOSSIDAE	*Quasipaa shini*	LC	Over-exploited	VU	EN
ANURA	DICROGLOSSIDAE	*Quasipaa spinosa*	LC	Over-exploited	VU	VU

Order	Family	Genus and species	1980 backcasted Red List category	Primary driver of change between 1980–2004	2004 backcasted Red List category	2022 GAA2 Red List category
ANURA	DICROGLOSSIDAE	*Quasipaa yei*	NT	Over-exploited	VU	VU
ANURA	ELEUTHERODACTYLIDAE	*Eleutherodactylus coqui*	LC	Disease	NT	LC
ANURA	ELEUTHERODACTYLIDAE	*Eleutherodactylus darlingtoni*	CR	Reduced-habitat	CR(PE)	CR(PE)
ANURA	ELEUTHERODACTYLIDAE	*Eleutherodactylus eneidae*	CR	Disease	CR(PE)	CR(PE)
ANURA	ELEUTHERODACTYLIDAE	*Eleutherodactylus glanduliferoides*	CR	Reduced-habitat	CR(PE)	CR(PE)
ANURA	ELEUTHERODACTYLIDAE	*Eleutherodactylus jamaicensis*	VU	Disease	CR	CR
ANURA	ELEUTHERODACTYLIDAE	*Eleutherodactylus jasperi*	CR	Disease	CR(PE)	CR(PE)
ANURA	ELEUTHERODACTYLIDAE	*Eleutherodactylus karlschmidti*	CR	Disease	CR(PE)	CR(PE)
ANURA	ELEUTHERODACTYLIDAE	*Eleutherodactylus limbensis*	CR	Disease	CR(PE)	CR(PE)
ANURA	ELEUTHERODACTYLIDAE	*Eleutherodactylus locustus*	EN	Disease	CR	EN
ANURA	ELEUTHERODACTYLIDAE	*Eleutherodactylus lucioi*	CR	Reduced-habitat	CR(PE)	CR(PE)
ANURA	ELEUTHERODACTYLIDAE	*Eleutherodactylus orcutti*	CR	Disease	CR(PE)	CR(PE)
ANURA	ELEUTHERODACTYLIDAE	*Eleutherodactylus richmondi*	EN	Disease	CR	EN
ANURA	ELEUTHERODACTYLIDAE	*Eleutherodactylus rucillensis*	CR	Disease	CR(PE)	CR(PE)
ANURA	ELEUTHERODACTYLIDAE	*Eleutherodactylus schmidti*	CR	Disease	CR(PE)	CR(PE)
ANURA	ELEUTHERODACTYLIDAE	*Eleutherodactylus symingtoni*	EN	Disease	CR	CR
ANURA	ELEUTHERODACTYLIDAE	*Eleutherodactylus unicolor*	VU	Climate change effects	CR	CR
ANURA	HEMIPHRACTIDAE	*Gastrotheca angustifrons*	LC	Disease	CR(PE)	CR(PE)
ANURA	HEMIPHRACTIDAE	*Gastrotheca antomia*	VU	Disease	CR(PE)	CR(PE)
ANURA	HEMIPHRACTIDAE	*Gastrotheca christiani*	EN	Reduced-habitat	CR	CR
ANURA	HEMIPHRACTIDAE	*Gastrotheca cornuta*	EN	Disease	CR	CR
ANURA	HEMIPHRACTIDAE	*Gastrotheca dendronastes*	LC	Disease	EN	EN
ANURA	HEMIPHRACTIDAE	*Gastrotheca nebulanastes*	LC	Disease	EN	EN
ANURA	HEMIPHRACTIDAE	*Gastrotheca phelloderma*	LC	Disease	VU	VU
ANURA	HEMIPHRACTIDAE	*Gastrotheca pseustes*	LC	Disease	EN	NT
ANURA	HEMIPHRACTIDAE	*Gastrotheca riobambae*	LC	Reduced-habitat	EN	VU
ANURA	HEMIPHRACTIDAE	*Hemiphractus bubalus*	LC	Reduced-habitat	NT	VU
ANURA	HEMIPHRACTIDAE	*Hemiphractus elioti*	VU	Reduced-habitat	CR	CR
ANURA	HEMIPHRACTIDAE	*Hemiphractus fasciatus*	NT	Reduced-habitat	VU	VU
ANURA	HYLIDAE	*Aplastodiscus flumineus*	LC	Disease	CR	CR(PE)
ANURA	HYLIDAE	*Aplastodiscus musicus*	VU	Disease	EN	CR
ANURA	HYLIDAE	*Boana buriti*	VU	Reduced-habitat	EN	EN
ANURA	HYLIDAE	*Boana claresignata*	CR	Disease	CR(PE)	CR(PE)
ANURA	HYLIDAE	*Boana clepsydra*	CR	Disease	CR(PE)	CR(PE)
ANURA	HYLIDAE	*Boana cymbalum*	CR	Disease	EX	EX
ANURA	HYLIDAE	*Boana freicanecae*	NT	Reduced-habitat	EN	EN

Order	Family	Genus and species	1980 backcasted Red List category	Primary driver of change between 1980–2004	2004 backcasted Red List category	2022 GAA2 Red List category
ANURA	HYLIDAE	*Boana heilprini*	NT	Reduced-habitat	VU	VU
ANURA	HYLIDAE	*Boana secedens*	LC	Reduced-habitat	NT	EN
ANURA	HYLIDAE	*Bokermannohyla izecksohni*	EN	Reduced-habitat	CR	CR
ANURA	HYLIDAE	*Bokermannohyla ravida*	VU	Reduced-habitat	EN	CR
ANURA	HYLIDAE	*Bokermannohyla sagarana*	NT	Reduced-habitat	EN	EN
ANURA	HYLIDAE	*Bromeliohyla bromeliacia*	LC	Disease	EN	LC
ANURA	HYLIDAE	*Dendropsophus nekronastes*	LC	Reduced-habitat	CR	CR
ANURA	HYLIDAE	*Dryophytes bocourti*	EN	Disease	CR(PE)	CR(PE)
ANURA	HYLIDAE	*Dryophytes flaviventris*	VU	Reduced-habitat	EN	EN
ANURA	HYLIDAE	*Dryophytes suweonensis*	VU	Reduced-habitat	EN	EN
ANURA	HYLIDAE	*Duellmanohyla uranochroa*	VU	Disease	CR	VU
ANURA	HYLIDAE	*Hyloscirtus armatus*	LC	Disease	VU	NT
ANURA	HYLIDAE	*Hyloscirtus chlorosteus*	CR	Reduced-habitat	CR(PE)	CR(PE)
ANURA	HYLIDAE	*Hyloscirtus colymba*	LC	Disease	CR	EN
ANURA	HYLIDAE	*Hyloscirtus condor*	LC	Reduced-habitat	NT	EN
ANURA	HYLIDAE	*Hyloscirtus hillisi*	LC	Reduced-habitat	VU	CR
ANURA	HYLIDAE	*Isthmohyla angustilineata*	VU	Disease	CR	CR
ANURA	HYLIDAE	*Isthmohyla calypsa*	EN	Disease	CR(PE)	CR(PE)
ANURA	HYLIDAE	*Isthmohyla debilis*	EN	Disease	CR	CR
ANURA	HYLIDAE	*Isthmohyla graceae*	EN	Disease	CR	CR
ANURA	HYLIDAE	*Isthmohyla pictipes*	VU	Disease	CR	CR
ANURA	HYLIDAE	*Isthmohyla rivularis*	VU	Disease	CR	EN
ANURA	HYLIDAE	*Isthmohyla tica*	VU	Disease	CR	CR
ANURA	HYLIDAE	*Megastomatohyla pellita*	EN	Disease	CR	CR
ANURA	HYLIDAE	*Myersiohyla liliae*	LC	Reduced-habitat	NT	EN
ANURA	HYLIDAE	*Osteopilus pulchrilineatus*	NT	Disease	VU	VU
ANURA	HYLIDAE	*Phyllodytes tuberculosus*	LC	Reduced-habitat	VU	VU
ANURA	HYLIDAE	*Plectrohyla calvata*	EN	Disease	CR	CR
ANURA	HYLIDAE	*Plectrohyla glandulosa*	VU	Disease	CR	CR
ANURA	HYLIDAE	*Plectrohyla guatemalensis*	LC	Disease	NT	NT
ANURA	HYLIDAE	*Plectrohyla hartwegi*	LC	Disease	EN	EN
ANURA	HYLIDAE	*Plectrohyla pokomchi*	EN	Disease	CR	EN
ANURA	HYLIDAE	*Plectrohyla tecunumani*	EN	Reduced-habitat	CR	CR
ANURA	HYLIDAE	*Ptychohyla hypomykter*	LC	Disease	VU	VU
ANURA	HYLIDAE	*Sarcohyla calvicollina*	CR	Disease	CR(PE)	CR(PE)
ANURA	HYLIDAE	*Sarcohyla celata*	EN	Disease	CR	NT

Order	Family	Genus and species	1980 backcasted Red List category	Primary driver of change between 1980–2004	2004 backcasted Red List category	2022 GAA2 Red List category
ANURA	HYLIDAE	*Sarcohyla crassa*	EN	Reduced-habitat	CR	CR
ANURA	HYLIDAE	*Sarcohyla cyanomma*	CR	Disease	CR(PE)	CR(PE)
ANURA	HYLIDAE	*Sarcohyla psarosema*	CR	Reduced-habitat	CR(PE)	CR(PE)
ANURA	HYLIDAE	*Sarcohyla sabrina*	CR	Disease	CR(PE)	CR(PE)
ANURA	HYLIDAE	*Sarcohyla siopela*	CR	Disease	CR(PE)	CR(PE)
ANURA	HYLIDAE	*Sarcohyla thorectes*	EN	Disease	CR	EN
ANURA	HYLIDAE	*Scinax cabralensis*	NT	Reduced-habitat	EN	EN
ANURA	HYLIDAE	*Scinax caldarum*	VU	Reduced-habitat	EN	EN
ANURA	HYLIDAE	*Scinax jolyi*	LC	Reduced-habitat	EN	EN
ANURA	HYLIDAE	*Triprion spinosus*	LC	Disease	NT	NT
ANURA	HYLODIDAE	*Crossodactylus boulengeri*	CR	Disease	CR(PE)	CR(PE)
ANURA	HYLODIDAE	*Crossodactylus cyclospinus*	NT	Reduced-habitat	EN	EN
ANURA	HYLODIDAE	*Crossodactylus dispar*	CR	Disease	CR(PE)	CR(PE)
ANURA	HYLODIDAE	*Crossodactylus franciscanus*	CR	Disease	CR(PE)	CR(PE)
ANURA	HYLODIDAE	*Crossodactylus grandis*	CR	Disease	CR(PE)	CR(PE)
ANURA	HYLODIDAE	*Hylodes glaber*	CR	Disease	CR(PE)	CR(PE)
ANURA	HYLODIDAE	*Hylodes magalhaesi*	LC	Reduced-habitat	EN	EN
ANURA	HYLODIDAE	*Hylodes mertensi*	CR	Disease	CR(PE)	CR(PE)
ANURA	HYLODIDAE	*Hylodes perere*	LC	Reduced-habitat	EN	EN
ANURA	HYLODIDAE	*Hylodes vanzolinii*	CR	Disease	CR(PE)	CR(PE)
ANURA	HYLODIDAE	*Phantasmarana boticariana*	LC	Reduced-habitat	EN	EN
ANURA	HYPEROLIIDAE	*Hyperolius cystocandicans*	VU	Reduced-habitat	EN	EN
ANURA	LEIOPELMATIDAE	*Leiopelma archeyi*	NT	Disease	CR	CR
ANURA	LEPTODACTYLIDAE	*Adenomera kweti*	NT	Reduced-habitat	EN	EN
ANURA	LEPTODACTYLIDAE	*Adenomera lutzi*	LC	Reduced-habitat	NT	EN
ANURA	LEPTODACTYLIDAE	*Adenomera phonotriccus*	LC	Reduced-habitat	CR	CR
ANURA	LEPTODACTYLIDAE	*Leptodactylus fallax*	EN	Disease	CR	CR
ANURA	LEPTODACTYLIDAE	*Leptodactylus laticeps*	LC	Over-exploited	NT	NT
ANURA	LEPTODACTYLIDAE	*Leptodactylus peritoaktites*	NT	Reduced-habitat	VU	EN
ANURA	LEPTODACTYLIDAE	*Leptodactylus silvanimbus*	EN	Reduced-habitat	CR	CR
ANURA	LEPTODACTYLIDAE	*Paratelmatobius lutzii*	CR	Disease	CR(PE)	CR(PE)
ANURA	LEPTODACTYLIDAE	*Pseudopaludicola ibisoroca*	LC	Reduced-habitat	VU	VU
ANURA	LEPTODACTYLIDAE	*Pseudopaludicola jazmynmcdonaldae*	NT	Reduced-habitat	EN	EN
ANURA	LIMNODYNASTIDAE	*Adelotus brevis*	LC	Disease	NT	LC
ANURA	LIMNODYNASTIDAE	*Heleioporus australiacus*	LC	Disease	EN	EN
ANURA	LIMNODYNASTIDAE	*Philoria frosti*	EN	Disease	CR	CR

Order	Family	Genus and species	1980 backcasted Red List category	Primary driver of change between 1980–2004	2004 backcasted Red List category	2022 GAA2 Red List category
ANURA	MEGOPHRYIDAE	*Leptobrachium boringii*	NT	Over-exploited	EN	EN
ANURA	MICROHYLIDAE	*Anilany helenae*	EN	Reduced-habitat	CR	CR
ANURA	MICROHYLIDAE	*Chiasmocleis papachibe*	LC	Reduced-habitat	NT	EN
ANURA	MICROHYLIDAE	*Chiasmocleis parkeri*	LC	Reduced-habitat	NT	EN
ANURA	MICROHYLIDAE	*Chiasmocleis quilombola*	NT	Reduced-habitat	VU	VU
ANURA	MICROHYLIDAE	*Chiasmocleis sapiranga*	NT	Reduced-habitat	EN	EN
ANURA	MICROHYLIDAE	*Choerophryne siegfriedi*	VU	Reduced-habitat	CR	CR
ANURA	MICROHYLIDAE	*Cophixalus neglectus*	VU	Climate change effects	EN	CR
ANURA	MICROHYLIDAE	*Cophixalus nubicola*	LC	Climate change effects	VU	VU
ANURA	MICROHYLIDAE	*Elachistocleis erythrogaster*	LC	Reduced-habitat	NT	EN
ANURA	MICROHYLIDAE	*Glyphoglossus molossus*	LC	Over-exploited	NT	NT
ANURA	MYOBATRACHIDAE	*Crinia flindersensis*	LC	Reduced-habitat	NT	NT
ANURA	MYOBATRACHIDAE	*Crinia riparia*	LC	Reduced-habitat	EN	EN
ANURA	MYOBATRACHIDAE	*Crinia tasmaniensis*	LC	Disease	NT	NT
ANURA	MYOBATRACHIDAE	*Mixophyes balbus*	LC	Disease	VU	VU
ANURA	MYOBATRACHIDAE	*Pseudophryne bibronii*	LC	Disease	NT	LC
ANURA	MYOBATRACHIDAE	*Pseudophryne corroboree*	VU	Disease	CR	CR
ANURA	MYOBATRACHIDAE	*Pseudophryne pengilleyi*	VU	Disease	EN	CR
ANURA	MYOBATRACHIDAE	*Rheobatrachus silus*	CR	Disease	EX	EX
ANURA	MYOBATRACHIDAE	*Rheobatrachus vitellinus*	EN	Disease	EX	EX
ANURA	MYOBATRACHIDAE	*Taudactylus acutirostris*	LC	Disease	CR(PE)	EX
ANURA	MYOBATRACHIDAE	*Taudactylus eungellensis*	LC	Disease	EN	EN
ANURA	MYOBATRACHIDAE	*Taudactylus rheophilus*	LC	Disease	CR(PE)	CR(PE)
ANURA	MYOBATRACHIDAE	*Uperoleia daviesae*	NT	Reduced-habitat	EN	EN
ANURA	MYOBATRACHIDAE	*Uperoleia martini*	NT	Climate change effects	VU	VU
ANURA	ODONTOPHRYNIDAE	*Proceratophrys huntingtoni*	LC	Reduced-habitat	VU	VU
ANURA	PELOBATIDAE	*Pelobates cultripes*	LC	Reduced-habitat	NT	VU
ANURA	PELOBATIDAE	*Pelobates varaldii*	VU	Reduced-habitat	EN	EN
ANURA	PELODRYADIDAE	*Litoria aurea*	NT	Disease	VU	NT
ANURA	PELODRYADIDAE	*Litoria booroolongensis*	VU	Disease	EN	EN
ANURA	PELODRYADIDAE	*Litoria dayi*	LC	Disease	EN	VU
ANURA	PELODRYADIDAE	*Litoria kroombitensis*	VU	Disease	CR	CR
ANURA	PELODRYADIDAE	*Litoria littlejohni*	VU	Disease	EN	EN
ANURA	PELODRYADIDAE	*Litoria lorica*	EN	Disease	CR	CR
ANURA	PELODRYADIDAE	*Litoria nannotis*	LC	Disease	EN	LC
ANURA	PELODRYADIDAE	*Litoria nyakalensis*	LC	Disease	EX	EX

Order	Family	Genus and species	1980 backcasted Red List category	Primary driver of change between 1980–2004	2004 backcasted Red List category	2022 GAA2 Red List category
ANURA	PELODRYADIDAE	*Litoria pearsoniana*	LC	Disease	NT	LC
ANURA	PELODRYADIDAE	*Litoria rheocola*	LC	Disease	EN	NT
ANURA	PELODRYADIDAE	*Litoria spenceri*	EN	Disease	CR	CR
ANURA	PHYLLOMEDUSIDAE	*Agalychnis annae*	NT	Disease	EN	VU
ANURA	PHYLLOMEDUSIDAE	*Agalychnis lemur*	LC	Disease	CR	CR
ANURA	PHYLLOMEDUSIDAE	*Phrynomedusa marginata*	CR	Disease	CR(PE)	CR(PE)
ANURA	PHYLLOMEDUSIDAE	*Phrynomedusa vanzolinii*	CR	Disease	CR(PE)	CR(PE)
ANURA	PTYCHADENIDAE	*Ptychadena newtoni*	VU	Reduced-habitat	EN	EN
ANURA	PYXICEPHALIDAE	*Amietia hymenopus*	LC	Disease	NT	NT
ANURA	PYXICEPHALIDAE	*Ericabatrachus baleensis*	VU	Disease	CR	CR
ANURA	PYXICEPHALIDAE	*Microbatrachella capensis*	EN	Reduced-habitat	CR	CR
ANURA	PYXICEPHALIDAE	*Strongylopus rhodesianus*	LC	Reduced-habitat	VU	VU
ANURA	RANIDAE	*Amolops hainanensis*	VU	Over-exploited	EN	EN
ANURA	RANIDAE	*Amolops loloensis*	NT	Reduced-habitat	VU	VU
ANURA	RANIDAE	*Lithobates capito*	NT	Reduced-habitat	VU	VU
ANURA	RANIDAE	*Lithobates megapoda*	LC	Reduced-habitat	NT	NT
ANURA	RANIDAE	*Lithobates tlaloci*	CR	Reduced-habitat	CR(PE)	CR(PE)
ANURA	RANIDAE	*Lithobates vibicarius*	VU	Disease	CR	EN
ANURA	RANIDAE	*Odorrana grahami*	NT	Over-exploited	VU	VU
ANURA	RANIDAE	*Odorrana hejiangensis*	NT	Over-exploited	VU	VU
ANURA	RANIDAE	*Odorrana jingdongensis*	LC	Over-exploited	VU	VU
ANURA	RANIDAE	*Pelophylax caralitanus*	LC	Reduced-habitat	NT	VU
ANURA	RANIDAE	*Pelophylax chosenicus*	LC	Reduced-habitat	NT	VU
ANURA	RANIDAE	*Pelophylax shqipericus*	NT	Reduced-habitat	VU	VU
ANURA	RANIDAE	*Rana asiatica*	LC	Reduced-habitat	VU	VU
ANURA	RANIDAE	*Rana dybowskii*	LC	Over-exploited	NT	NT
ANURA	RANIDAE	*Rana iberica*	LC	Reduced-habitat	NT	VU
ANURA	RANIDAE	*Rana latastei*	NT	Reduced-habitat	VU	VU
ANURA	RHINODERMATIDAE	*Rhinoderma darwinii*	NT	Disease	VU	EN
ANURA	RHINODERMATIDAE	*Rhinoderma rufum*	CR	Reduced-habitat	CR(PE)	CR(PE)
ANURA	TELMATOBIIDAE	*Telmatobius atacamensis*	EN	Disease	CR	CR
ANURA	TELMATOBIIDAE	*Telmatobius bolivianus*	VU	Disease	CR	CR(PE)
ANURA	TELMATOBIIDAE	*Telmatobius ceiorum*	CR	Disease	CR(PE)	CR(PE)
ANURA	TELMATOBIIDAE	*Telmatobius cirrhacelis*	EN	Disease	CR(PE)	CR(PE)
ANURA	TELMATOBIIDAE	*Telmatobius culeus*	VU	Numerous	EN	EN
ANURA	TELMATOBIIDAE	*Telmatobius edaphonastes*	EN	Disease	CR	CR(PE)

Order	Family	Genus and species	1980 backcasted Red List category	Primary driver of change between 1980–2004	2004 backcasted Red List category	2022 GAA2 Red List category
ANURA	TELMATOBIIDAE	*Telmatobius espadai*	EN	Disease	CR	CR(PE)
ANURA	TELMATOBIIDAE	*Telmatobius hintoni*	LC	Disease	NT	VU
ANURA	TELMATOBIIDAE	*Telmatobius jelskii*	LC	Numerous	NT	NT
ANURA	TELMATOBIIDAE	*Telmatobius laticeps*	EN	Disease	CR(PE)	CR(PE)
ANURA	TELMATOBIIDAE	*Telmatobius marmoratus*	LC	Over-exploited	EN	EN
ANURA	TELMATOBIIDAE	*Telmatobius mendelsoni*	LC	Disease	VU	CR(PE)
ANURA	TELMATOBIIDAE	*Telmatobius niger*	VU	Disease	CR(PE)	CR(PE)
ANURA	TELMATOBIIDAE	*Telmatobius sanborni*	VU	Disease	CR	CR
ANURA	TELMATOBIIDAE	*Telmatobius sibiricus*	EN	Disease	CR	CR(PE)
ANURA	TELMATOBIIDAE	*Telmatobius simonsi*	LC	Disease	CR	CR
ANURA	TELMATOBIIDAE	*Telmatobius timens*	VU	Disease	CR	CR
ANURA	TELMATOBIIDAE	*Telmatobius vellardi*	EN	Disease	CR(PE)	CR(PE)
ANURA	TELMATOBIIDAE	*Telmatobius verrucosus*	VU	Disease	CR	CR
ANURA	TELMATOBIIDAE	*Telmatobius yuracare*	EN	Disease	CR	CR
ANURA	TELMATOBIIDAE	*Telmatobius zapahuirensis*	VU	Reduced-habitat	EN	EN
CAUDATA	AMBYSTOMATIDAE	*Ambystoma cingulatum*	NT	Reduced-habitat	VU	EN
CAUDATA	AMBYSTOMATIDAE	*Ambystoma granulosum*	VU	Disease	EN	EN
CAUDATA	CRYPTOBRANCHIDAE	*Andrias davidianus*	NT	Over-exploited	CR	CR
CAUDATA	CRYPTOBRANCHIDAE	*Andrias sligoi*	NT	Over-exploited	CR	CR
CAUDATA	HYNOBIIDAE	*Batrachuperus karlschmidti*	LC	Over-exploited	VU	VU
CAUDATA	HYNOBIIDAE	*Batrachuperus pinchonii*	LC	Over-exploited	VU	VU
CAUDATA	HYNOBIIDAE	*Batrachuperus yenyuanensis*	VU	Over-exploited	EN	EN
CAUDATA	HYNOBIIDAE	*Ranodon sibiricus*	VU	Reduced-habitat	EN	EN
CAUDATA	PLETHODONTIDAE	*Batrachoseps minor*	NT	Climate change effects	EN	EN
CAUDATA	PLETHODONTIDAE	*Bolitoglossa dofleini*	LC	Reduced-habitat	NT	NT
CAUDATA	PLETHODONTIDAE	*Bolitoglossa nussbaumi*	CR	Reduced-habitat	CR(PE)	CR(PE)
CAUDATA	PLETHODONTIDAE	*Bolitoglossa pesrubra*	LC	Unknown	VU	LC
CAUDATA	PLETHODONTIDAE	*Chiropterotriton chiropterus*	EN	Reduced-habitat	CR	CR
CAUDATA	PLETHODONTIDAE	*Chiropterotriton magnipes*	EN	Disease	CR	EN
CAUDATA	PLETHODONTIDAE	*Desmognathus abditus*	NT	Reduced-habitat	VU	VU
CAUDATA	PLETHODONTIDAE	*Isthmura gigantea*	VU	Disease	EN	EN
CAUDATA	PLETHODONTIDAE	*Isthmura naucampatepetl*	EN	Reduced-habitat	CR	CR
CAUDATA	PLETHODONTIDAE	*Oedipina gracilis*	VU	Reduced-habitat	EN	EN
CAUDATA	PLETHODONTIDAE	*Oedipina petiola*	CR	Reduced-habitat	CR(PE)	CR(PE)
CAUDATA	PLETHODONTIDAE	*Oedipina uniformis*	LC	Unknown	NT	LC
CAUDATA	PLETHODONTIDAE	*Pseudoeurycea aquatica*	CR	Reduced-habitat	CR(PE)	CR(PE)

Order	Family	Genus and species	1980 backcasted Red List category	Primary driver of change between 1980–2004	2004 backcasted Red List category	2022 GAA2 Red List category
CAUDATA	PLETHODONTIDAE	*Pseudoeurycea brunnata*	EN	Disease	CR(PE)	CR(PE)
CAUDATA	PLETHODONTIDAE	*Pseudoeurycea exspectata*	CR	Reduced-habitat	CR(PE)	EX
CAUDATA	PLETHODONTIDAE	*Pseudoeurycea goebeli*	EN	Disease	CR	CR
CAUDATA	PLETHODONTIDAE	*Pseudoeurycea rex*	LC	Disease	CR	VU
CAUDATA	PLETHODONTIDAE	*Pseudoeurycea smithi*	EN	Disease	CR	CR
CAUDATA	PLETHODONTIDAE	*Pseudoeurycea unguidentis*	CR	Disease	CR(PE)	CR(PE)
CAUDATA	PLETHODONTIDAE	*Speleomantes supramontis*	NT	Reduced-habitat	EN	EN
CAUDATA	PLETHODONTIDAE	*Thorius longicaudus*	EN	Disease	CR	CR(PE)
CAUDATA	SALAMANDRIDAE	*Chioglossa lusitanica*	LC	Reduced-habitat	NT	NT
CAUDATA	SALAMANDRIDAE	*Taricha rivularis*	LC	Reduced-habitat	VU	VU
CAUDATA	SALAMANDRIDAE	*Triturus pygmaeus*	LC	Reduced-habitat	NT	NT
CAUDATA	SALAMANDRIDAE	*Tylototriton asperrimus*	LC	Over-exploited	NT	NT
CAUDATA	SALAMANDRIDAE	*Tylototriton shanjing*	LC	Over-exploited	NT	VU
CAUDATA	SALAMANDRIDAE	*Tylototriton wenxianensis*	LC	Over-exploited	VU	VU
GYMNOPHIONA	HERPELIDAE	*Boulengerula denhardti*	EN	Reduced-habitat	CR	CR
GYMNOPHIONA	RHINATREMATIDAE	*Rhinatrema shiv*	LC	Reduced-habitat	NT	EN

Table 3a. Species that deteriorated in status between 1980–2004 categorized by primary drivers of decline.

Supplementary Table 3b

Order	Family	Genus and species	1980 backcasted Red List category	2004 backcasted Red List category	Primary driver of change between 2004–2022	2022 GAA2 Red List category
ANURA	ALSODIDAE	*Alsodes pehuenche*	VU	VU	Reduced-habitat	CR
ANURA	ALYTIDAE	*Alytes dickhilleni*	NT	VU	Disease	EN
ANURA	ALYTIDAE	*Alytes maurus*	NT	NT	Disease	EN
ANURA	AROMOBATIDAE	*Allobates chalcopis*	VU	VU	Climate change effects	CR
ANURA	AROMOBATIDAE	*Allobates goianus*	LC	LC	Reduced-habitat	NT
ANURA	AROMOBATIDAE	*Allobates mcdiarmidi*	NT	CR	Disease	CR(PE)
ANURA	AROMOBATIDAE	*Allobates pacaas*	LC	LC	Climate change effects	VU
ANURA	AROMOBATIDAE	*Allobates undulatus*	LC	LC	Climate change effects	VU
ANURA	AROMOBATIDAE	*Anomaloglossus apiau*	LC	LC	Climate change effects	NT
ANURA	AROMOBATIDAE	*Anomaloglossus ayarzaguenai*	LC	LC	Climate change effects	VU
ANURA	AROMOBATIDAE	*Anomaloglossus blanci*	LC	LC	Disease	EN
ANURA	AROMOBATIDAE	*Anomaloglossus breweri*	LC	LC	Climate change effects	NT
ANURA	AROMOBATIDAE	*Anomaloglossus degranvillei*	LC	LC	Disease	CR
ANURA	AROMOBATIDAE	*Anomaloglossus dewynteri*	LC	LC	Disease	CR
ANURA	AROMOBATIDAE	*Anomaloglossus guanayensis*	LC	LC	Climate change effects	NT
ANURA	AROMOBATIDAE	*Anomaloglossus kaiei*	LC	NT	Reduced-habitat	EN
ANURA	AROMOBATIDAE	*Anomaloglossus moffetti*	LC	LC	Climate change effects	NT
ANURA	AROMOBATIDAE	*Anomaloglossus praderioi*	NT	NT	Reduced-habitat	EN
ANURA	AROMOBATIDAE	*Anomaloglossus roraima*	NT	NT	Reduced-habitat	EN
ANURA	AROMOBATIDAE	*Anomaloglossus rufulus*	LC	LC	Climate change effects	NT
ANURA	AROMOBATIDAE	*Anomaloglossus shrevei*	LC	LC	Climate change effects	NT
ANURA	ARTHROLEPTIDAE	*Arthroleptis krokosua*	EN	EN	Reduced-habitat	CR
ANURA	BATRACHYLIDAE	*Atelognathus patagonicus*	EN	EN	Climate change effects	CR
ANURA	BRACHYCEPHALIDAE	*Brachycephalus brunneus*	LC	LC	Climate change effects	VU
ANURA	BRACHYCEPHALIDAE	*Brachycephalus coloratus*	LC	LC	Climate change effects	VU
ANURA	BRACHYCEPHALIDAE	*Brachycephalus crispus*	LC	LC	Climate change effects	VU
ANURA	BRACHYCEPHALIDAE	*Brachycephalus izecksohni*	LC	LC	Climate change effects	VU
ANURA	BRACHYCEPHALIDAE	*Brachycephalus leopardus*	LC	LC	Climate change effects	VU
ANURA	BRACHYCEPHALIDAE	*Brachycephalus pombali*	LC	LC	Climate change effects	VU
ANURA	BRACHYCEPHALIDAE	*Brachycephalus verrucosus*	LC	LC	Climate change effects	VU
ANURA	BRACHYCEPHALIDAE	*Ischnocnema bocaina*	LC	LC	Climate change effects	VU
ANURA	BRACHYCEPHALIDAE	*Ischnocnema paranaensis*	LC	LC	Climate change effects	VU
ANURA	BRACHYCEPHALIDAE	*Ischnocnema parnaso*	LC	LC	Climate change effects	VU
ANURA	BUFONIDAE	*Altiphrynoides osgoodi*	CR	CR	Disease	CR(PE)

Order	Family	Genus and species	1980 backcasted Red List category	2004 backcasted Red List category	Primary driver of change between 2004–2022	2022 GAA2 Red List category
ANURA	BUFONIDAE	*Ansonia guibei*	VU	VU	Reduced-habitat	CR
ANURA	BUFONIDAE	*Atelopus certus*	EN	EN	Disease	CR
ANURA	BUFONIDAE	*Atelopus ebenoides*	EN	CR	Disease	CR(PE)
ANURA	BUFONIDAE	*Atelopus erythropus*	LC	CR	Disease	CR(PE)
ANURA	BUFONIDAE	*Atelopus eusebianus*	EN	CR	Disease	CR(PE)
ANURA	BUFONIDAE	*Atelopus farci*	CR	CR	Disease	CR(PE)
ANURA	BUFONIDAE	*Atelopus glyphus*	LC	LC	Disease	CR
ANURA	BUFONIDAE	*Atelopus limosus*	EN	EN	Disease	CR
ANURA	BUFONIDAE	*Atelopus palmatus*	EN	EN	Reduced-habitat	CR
ANURA	BUFONIDAE	*Atelopus peruensis*	NT	CR	Disease	CR(PE)
ANURA	BUFONIDAE	*Atelopus pinangoi*	EN	CR	Disease	CR(PE)
ANURA	BUFONIDAE	*Atelopus simulatus*	VU	CR	Disease	CR(PE)
ANURA	BUFONIDAE	*Atelopus tricolor*	LC	EN	Disease	CR
ANURA	BUFONIDAE	*Atelopus zeteki*	EN	CR	Disease	CR(PEW)
ANURA	BUFONIDAE	*Duttaphrynus noellerti*	EN	EN	Numerous	CR
ANURA	BUFONIDAE	*Incilius peripatetes*	CR	CR	Disease	CR(PE)
ANURA	BUFONIDAE	*Melanophryniscus xanthostomus*	LC	LC	Reduced-habitat	NT
ANURA	BUFONIDAE	*Metaphryniscus sosai*	LC	LC	Climate change effects	NT
ANURA	BUFONIDAE	*Nannophryne cophotis*	NT	CR	Disease	CR(PE)
ANURA	BUFONIDAE	*Nectophrynoides asperginis*	VU	CR	Disease	EW
ANURA	BUFONIDAE	*Oreophrynella cryptica*	LC	LC	Climate change effects	NT
ANURA	BUFONIDAE	*Oreophrynella huberi*	LC	LC	Climate change effects	VU
ANURA	BUFONIDAE	*Oreophrynella macconnelli*	NT	NT	Reduced-habitat	VU
ANURA	BUFONIDAE	*Oreophrynella nigra*	LC	LC	Climate change effects	VU
ANURA	BUFONIDAE	*Oreophrynella quelchii*	LC	LC	Reduced-habitat	EN
ANURA	BUFONIDAE	*Oreophrynella seegobini*	LC	LC	Climate change effects	VU
ANURA	BUFONIDAE	*Oreophrynella vasquezi*	LC	LC	Climate change effects	VU
ANURA	BUFONIDAE	*Oreophrynella weiassipuensis*	LC	LC	Climate change effects	VU
ANURA	BUFONIDAE	*Osornophryne cofanorum*	VU	VU	Reduced-habitat	CR
ANURA	BUFONIDAE	*Peltophryne longinasus*	EN	EN	Climate change effects	CR
ANURA	BUFONIDAE	*Rhaebo blombergi*	NT	VU	Reduced-habitat	CR
ANURA	BUFONIDAE	*Rhinella atacamensis*	LC	LC	Climate change effects	VU
ANURA	BUFONIDAE	*Rhinella sebbeni*	LC	LC	Reduced-habitat	NT
ANURA	BUFONIDAE	*Werneria bambutensis*	EN	EN	Disease	CR
ANURA	BUFONIDAE	*Werneria mertensiana*	EN	EN	Disease	CR
ANURA	BUFONIDAE	*Werneria tandyi*	EN	EN	Disease	CR

Order	Family	Genus and species	1980 backcasted Red List category	2004 backcasted Red List category	Primary driver of change between 2004–2022	2022 GAA2 Red List category
ANURA	BUFONIDAE	*Wolterstorffina parvipalmata*	VU	VU	Disease	CR
ANURA	CENTROLENIDAE	*Centrolene buckleyi*	LC	VU	Reduced-habitat	CR
ANURA	CENTROLENIDAE	*Centrolene condor*	LC	NT	Reduced-habitat	EN
ANURA	CENTROLENIDAE	*Cochranella duidaeana*	LC	LC	Climate change effects	VU
ANURA	CENTROLENIDAE	*Cochranella mache*	LC	LC	Reduced-habitat	NT
ANURA	CENTROLENIDAE	*Cochranella riveroi*	LC	LC	Climate change effects	VU
ANURA	CENTROLENIDAE	*Hyalinobatrachium pellucidum*	LC	LC	Reduced-habitat	NT
ANURA	CENTROLENIDAE	*Nymphargus bejaranoi*	LC	VU	Disease	EN
ANURA	CENTROLENIDAE	*Nymphargus colomai*	LC	NT	Reduced-habitat	EN
ANURA	CENTROLENIDAE	*Nymphargus lindae*	LC	NT	Reduced-habitat	EN
ANURA	CENTROLENIDAE	*Nymphargus siren*	VU	VU	Reduced-habitat	EN
ANURA	CENTROLENIDAE	*Nymphargus truebae*	LC	CR	Disease	CR(PE)
ANURA	CENTROLENIDAE	*Rulyrana mcdiarmidi*	LC	LC	Reduced-habitat	NT
ANURA	CERATOBATRACHIDAE	*Cornufer gilliardi*	LC	LC	Reduced-habitat	NT
ANURA	CONRAUIDAE	*Conraua derooi*	EN	EN	Reduced-habitat	CR
ANURA	CRAUGASTORIDAE	*Ceuthomantis aracamuni*	LC	LC	Climate change effects	VU
ANURA	CRAUGASTORIDAE	*Ceuthomantis duellmani*	LC	LC	Climate change effects	NT
ANURA	CRAUGASTORIDAE	*Craugastor aphanus*	VU	VU	Climate change effects	EN
ANURA	CRAUGASTORIDAE	*Craugastor chingopetaca*	LC	LC	Climate change effects	VU
ANURA	CRAUGASTORIDAE	*Craugastor evanesco*	VU	VU	Disease	CR
ANURA	CRAUGASTORIDAE	*Craugastor inachus*	EN	EN	Climate change effects	CR
ANURA	CRAUGASTORIDAE	*Craugastor matudai*	VU	VU	Reduced-habitat	EN
ANURA	CRAUGASTORIDAE	*Craugastor punctariolus*	VU	VU	Disease	CR(PE)
ANURA	CRAUGASTORIDAE	*Dischidodactylus colonnelloi*	LC	LC	Climate change effects	NT
ANURA	CRAUGASTORIDAE	*Dischidodactylus duidensis*	LC	LC	Climate change effects	NT
ANURA	CRAUGASTORIDAE	*Holoaden luederwaldti*	LC	LC	Climate change effects	NT
ANURA	CRAUGASTORIDAE	*Lynchius simmonsi*	LC	NT	Reduced-habitat	EN
ANURA	CRAUGASTORIDAE	*Microkayla harveyi*	VU	VU	Reduced-habitat	CR
ANURA	CRAUGASTORIDAE	*Noblella lochites*	LC	NT	Reduced-habitat	EN
ANURA	CRAUGASTORIDAE	*Pristimantis abakapa*	LC	LC	Climate change effects	NT
ANURA	CRAUGASTORIDAE	*Pristimantis almendariz*	LC	LC	Reduced-habitat	CR
ANURA	CRAUGASTORIDAE	*Pristimantis andinogigas*	VU	VU	Climate change effects	CR
ANURA	CRAUGASTORIDAE	*Pristimantis ardyae*	LC	LC	Climate change effects	VU
ANURA	CRAUGASTORIDAE	*Pristimantis auricarens*	LC	LC	Climate change effects	NT
ANURA	CRAUGASTORIDAE	*Pristimantis barrigai*	LC	VU	Reduced-habitat	CR
ANURA	CRAUGASTORIDAE	*Pristimantis boconoensis*	LC	LC	Climate change effects	VU

Order	Family	Genus and species	1980 backcasted Red List category	2004 backcasted Red List category	Primary driver of change between 2004–2022	2022 GAA2 Red List category
ANURA	CRAUGASTORIDAE	*Pristimantis briceni*	NT	NT	Reduced-habitat	EN
ANURA	CRAUGASTORIDAE	*Pristimantis cantitans*	LC	LC	Climate change effects	NT
ANURA	CRAUGASTORIDAE	*Pristimantis chocoensis*	EN	EN	Reduced-habitat	CR
ANURA	CRAUGASTORIDAE	*Pristimantis citriogaster*	LC	LC	Reduced-habitat	EN
ANURA	CRAUGASTORIDAE	*Pristimantis flabellidiscus*	LC	LC	Climate change effects	VU
ANURA	CRAUGASTORIDAE	*Pristimantis guaiquinimensis*	LC	LC	Reduced-habitat	NT
ANURA	CRAUGASTORIDAE	*Pristimantis imthurni*	VU	VU	Climate change effects	CR
ANURA	CRAUGASTORIDAE	*Pristimantis jabonensis*	LC	LC	Climate change effects	VU
ANURA	CRAUGASTORIDAE	*Pristimantis jamescameroni*	VU	VU	Climate change effects	CR
ANURA	CRAUGASTORIDAE	*Pristimantis kareliae*	VU	VU	Reduced-habitat	CR
ANURA	CRAUGASTORIDAE	*Pristimantis lasalleorum*	NT	NT	Reduced-habitat	EN
ANURA	CRAUGASTORIDAE	*Pristimantis marahuaka*	LC	LC	Climate change effects	NT
ANURA	CRAUGASTORIDAE	*Pristimantis minimus*	LC	NT	Reduced-habitat	EN
ANURA	CRAUGASTORIDAE	*Pristimantis muchimuk*	LC	LC	Climate change effects	VU
ANURA	CRAUGASTORIDAE	*Pristimantis multicolor*	LC	LC	Climate change effects	VU
ANURA	CRAUGASTORIDAE	*Pristimantis museosus*	NT	NT	Disease	VU
ANURA	CRAUGASTORIDAE	*Pristimantis nangaritza*	LC	VU	Reduced-habitat	CR
ANURA	CRAUGASTORIDAE	*Pristimantis orcesi*	LC	LC	Reduced-habitat	VU
ANURA	CRAUGASTORIDAE	*Pristimantis ornatissimus*	VU	VU	Reduced-habitat	EN
ANURA	CRAUGASTORIDAE	*Pristimantis paquishae*	LC	VU	Reduced-habitat	CR
ANURA	CRAUGASTORIDAE	*Pristimantis parvillus*	LC	NT	Reduced-habitat	VU
ANURA	CRAUGASTORIDAE	*Pristimantis pluvian*	LC	LC	Reduced-habitat	NT
ANURA	CRAUGASTORIDAE	*Pristimantis pruinatus*	LC	LC	Climate change effects	VU
ANURA	CRAUGASTORIDAE	*Pristimantis rhigophilus*	LC	LC	Climate change effects	VU
ANURA	CRAUGASTORIDAE	*Pristimantis sarisarinama*	LC	LC	Climate change effects	NT
ANURA	CRAUGASTORIDAE	*Pristimantis satagius*	NT	NT	Reduced-habitat	EN
ANURA	CRAUGASTORIDAE	*Pristimantis yanezi*	LC	LC	Climate change effects	NT
ANURA	CRAUGASTORIDAE	*Pristimantis yantzaza*	LC	NT	Reduced-habitat	EN
ANURA	CRAUGASTORIDAE	*Pristimantis yaviensis*	LC	LC	Climate change effects	NT
ANURA	CRAUGASTORIDAE	*Pristimantis yuruaniensis*	LC	LC	Climate change effects	VU
ANURA	CRAUGASTORIDAE	*Strabomantis bufoniformis*	LC	VU	Disease	EN
ANURA	CRAUGASTORIDAE	*Yunganastes fraudator*	LC	NT	Disease	VU
ANURA	CRAUGASTORIDAE	*Yunganastes pluvicanorus*	LC	NT	Disease	VU
ANURA	CYCLORAMPHIDAE	*Cycloramphus bandeirensis*	LC	LC	Climate change effects	VU
ANURA	CYCLORAMPHIDAE	*Cycloramphus organensis*	LC	LC	Climate change effects	VU
ANURA	DENDROBATIDAE	*Ameerega boehmei*	LC	LC	Reduced-habitat	EN

Order	Family	Genus and species	1980 backcasted Red List category	2004 backcasted Red List category	Primary driver of change between 2004–2022	2022 GAA2 Red List category
ANURA	DENDROBATIDAE	*Ameerega boliviana*	LC	LC	Reduced-habitat	NT
ANURA	DENDROBATIDAE	*Andinobates abditus*	CR	CR	Reduced-habitat	CR(PE)
ANURA	DENDROBATIDAE	*Andinobates viridis*	EN	CR	Disease	CR(PE)
ANURA	DENDROBATIDAE	*Colostethus latinasus*	LC	LC	Disease	CR
ANURA	DENDROBATIDAE	*Hyloxalus infraguttatus*	LC	NT	Reduced-habitat	VU
ANURA	DENDROBATIDAE	*Hyloxalus mystax*	LC	NT	Reduced-habitat	EN
ANURA	DENDROBATIDAE	*Oophaga andresi*	LC	LC	Reduced-habitat	EN
ANURA	DENDROBATIDAE	*Paruwrobates erythromos*	EN	EN	Reduced-habitat	CR
ANURA	DENDROBATIDAE	*Silverstoneia nubicola*	LC	NT	Disease	VU
ANURA	DICROGLOSSIDAE	*Quasipaa shini*	LC	VU	Over-exploited	EN
ANURA	ELEUTHERODACTYLIDAE	*Eleutherodactylus gryllus*	EN	EN	Climate change effects	CR
ANURA	ELEUTHERODACTYLIDAE	*Eleutherodactylus turquinensis*	EN	EN	Disease	CR
ANURA	HEMIPHRACTIDAE	*Hemiphractus bubalus*	LC	NT	Reduced-habitat	VU
ANURA	HEMIPHRACTIDAE	*Hemiphractus kaylockae*	NT	NT	Disease	EN
ANURA	HEMIPHRACTIDAE	*Hemiphractus panamensis*	EN	EN	Disease	CR
ANURA	HEMIPHRACTIDAE	*Stefania breweri*	LC	LC	Climate change effects	VU
ANURA	HEMIPHRACTIDAE	*Stefania ginesi*	LC	LC	Climate change effects	NT
ANURA	HEMIPHRACTIDAE	*Stefania goini*	LC	LC	Climate change effects	NT
ANURA	HEMIPHRACTIDAE	*Stefania marahuaquensis*	LC	LC	Climate change effects	NT
ANURA	HEMIPHRACTIDAE	*Stefania oculosa*	LC	LC	Climate change effects	VU
ANURA	HEMIPHRACTIDAE	*Stefania percristata*	LC	LC	Climate change effects	VU
ANURA	HEMIPHRACTIDAE	*Stefania riae*	LC	LC	Climate change effects	NT
ANURA	HEMIPHRACTIDAE	*Stefania riveroi*	LC	LC	Climate change effects	VU
ANURA	HEMIPHRACTIDAE	*Stefania satelles*	LC	LC	Climate change effects	NT
ANURA	HEMIPHRACTIDAE	*Stefania schuberti*	LC	LC	Climate change effects	NT
ANURA	HYLIDAE	*Aplastodiscus flumineus*	LC	CR	Disease	CR(PE)
ANURA	HYLIDAE	*Aplastodiscus musicus*	VU	EN	Disease	CR
ANURA	HYLIDAE	*Atlantihyla panchoi*	EN	EN	Reduced-habitat	CR
ANURA	HYLIDAE	*Boana rhythmica*	LC	LC	Climate change effects	NT
ANURA	HYLIDAE	*Boana secedens*	LC	NT	Reduced-habitat	EN
ANURA	HYLIDAE	*Bokermannohyla carvalhoi*	LC	LC	Climate change effects	NT
ANURA	HYLIDAE	*Bokermannohyla flavopicta*	LC	LC	Climate change effects	NT
ANURA	HYLIDAE	*Bokermannohyla ravida*	VU	EN	Reduced-habitat	CR
ANURA	HYLIDAE	*Dendropsophus pelidnus*	LC	LC	Reduced-habitat	NT
ANURA	HYLIDAE	*Dendropsophus rozenmani*	LC	LC	Reduced-habitat	EN
ANURA	HYLIDAE	*Ecnomiohyla rabborum*	CR	CR	Disease	CR(PE)

Order	Family	Genus and species	1980 backcasted Red List category	2004 backcasted Red List category	Primary driver of change between 2004–2022	2022 GAA2 Red List category
ANURA	HYLIDAE	*Hyloscirtus condor*	LC	NT	Reduced-habitat	EN
ANURA	HYLIDAE	*Hyloscirtus hillisi*	LC	VU	Reduced-habitat	CR
ANURA	HYLIDAE	*Myersiohyla aromatica*	LC	LC	Climate change effects	VU
ANURA	HYLIDAE	*Myersiohyla chamaeleo*	LC	LC	Climate change effects	NT
ANURA	HYLIDAE	*Myersiohyla inparquesi*	LC	LC	Climate change effects	NT
ANURA	HYLIDAE	*Myersiohyla liliae*	LC	NT	Reduced-habitat	EN
ANURA	HYLIDAE	*Myersiohyla loveridgei*	LC	LC	Climate change effects	NT
ANURA	HYLIDAE	*Plectrohyla acanthodes*	VU	VU	Reduced-habitat	EN
ANURA	HYLIDAE	*Tepuihyla aecii*	LC	LC	Climate change effects	NT
ANURA	HYLIDAE	*Tepuihyla luteolabris*	LC	LC	Climate change effects	NT
ANURA	HYLIDAE	*Tepuihyla obscura*	NT	NT	Reduced-habitat	EN
ANURA	HYLODIDAE	*Hylodes dactylocinus*	LC	LC	Reduced-habitat	EN
ANURA	HYLODIDAE	*Hylodes ornatus*	LC	LC	Climate change effects	VU
ANURA	LEPTODACTYLIDAE	*Adenomera cotuba*	LC	LC	Reduced-habitat	NT
ANURA	LEPTODACTYLIDAE	*Adenomera lutzi*	LC	NT	Reduced-habitat	EN
ANURA	LEPTODACTYLIDAE	*Crossodactylodes septentrionalis*	LC	LC	Climate change effects	VU
ANURA	LEPTODACTYLIDAE	*Leptodactylus marambaiae*	LC	LC	Climate change effects	NT
ANURA	LEPTODACTYLIDAE	*Leptodactylus peritoaktites*	NT	VU	Reduced-habitat	EN
ANURA	LEPTODACTYLIDAE	*Physalaemus erythros*	LC	LC	Climate change effects	NT
ANURA	LEPTODACTYLIDAE	*Physalaemus evangelistai*	LC	LC	Climate change effects	NT
ANURA	LEPTODACTYLIDAE	*Pleurodema marmoratum*	LC	LC	Disease	VU
ANURA	MEGOPHRYIDAE	*Megophrys fansipanensis*	VU	VU	Reduced-habitat	EN
ANURA	MICROHYLIDAE	*Barygenys apodasta*	LC	LC	Reduced-habitat	VU
ANURA	MICROHYLIDAE	*Chiasmocleis altomontana*	LC	LC	Climate change effects	VU
ANURA	MICROHYLIDAE	*Chiasmocleis papachibe*	LC	NT	Reduced-habitat	EN
ANURA	MICROHYLIDAE	*Chiasmocleis parkeri*	LC	NT	Reduced-habitat	EN
ANURA	MICROHYLIDAE	*Cophixalus aenigma*	NT	NT	Climate change effects	EN
ANURA	MICROHYLIDAE	*Cophixalus amabilis*	LC	LC	Reduced-habitat	VU
ANURA	MICROHYLIDAE	*Cophixalus clapporum*	LC	LC	Reduced-habitat	VU
ANURA	MICROHYLIDAE	*Cophixalus concinnus*	VU	VU	Climate change effects	CR
ANURA	MICROHYLIDAE	*Cophixalus hosmeri*	NT	NT	Climate change effects	EN
ANURA	MICROHYLIDAE	*Cophixalus mcdonaldi*	VU	VU	Climate change effects	CR
ANURA	MICROHYLIDAE	*Cophixalus monticola*	VU	VU	Climate change effects	CR
ANURA	MICROHYLIDAE	*Cophixalus neglectus*	VU	EN	Climate change effects	CR
ANURA	MICROHYLIDAE	*Cophixalus sphagnicola*	LC	LC	Reduced-habitat	EN
ANURA	MICROHYLIDAE	*Elachistocleis erythrogaster*	LC	NT	Reduced-habitat	EN

Order	Family	Genus and species	1980 backcasted Red List category	2004 backcasted Red List category	Primary driver of change between 2004–2022	2022 GAA2 Red List category
ANURA	MICROHYLIDAE	*Mantophryne insignis*	LC	LC	Reduced-habitat	VU
ANURA	MICROHYLIDAE	*Oreophryne phoebe*	LC	LC	Reduced-habitat	VU
ANURA	MICROHYLIDAE	*Sphenophryne rhododactyla*	LC	LC	Reduced-habitat	VU
ANURA	MICROHYLIDAE	*Uperodon montanus*	LC	LC	Reduced-habitat	NT
ANURA	MYOBATRACHIDAE	*Assa darlingtoni*	LC	LC	Climate change effects	VU
ANURA	MYOBATRACHIDAE	*Assa wollumbin*	VU	VU	Climate change effects	CR
ANURA	MYOBATRACHIDAE	*Pseudophryne pengilleyi*	VU	EN	Disease	CR
ANURA	MYOBATRACHIDAE	*Uperoleia tyleri*	LC	LC	Climate change effects	NT
ANURA	PELOBATIDAE	*Pelobates cultripes*	LC	NT	Reduced-habitat	VU
ANURA	PELODRYADIDAE	*Litoria burrowsi*	LC	LC	Disease	NT
ANURA	PELODRYADIDAE	*Litoria castanea*	CR	CR	Climate change effects	CR(PE)
ANURA	PELODRYADIDAE	*Litoria cooloolensis*	LC	LC	Climate change effects	NT
ANURA	PETROPEDETIDAE	*Petropedetes perreti*	EN	EN	Disease	CR
ANURA	PHRYNOBATRACHIDAE	*Phrynobatrachus afiabirago*	EN	EN	Reduced-habitat	CR
ANURA	PHRYNOBATRACHIDAE	*Phrynobatrachus jimzimkusi*	VU	VU	Disease	CR
ANURA	PHRYNOBATRACHIDAE	*Phrynobatrachus manengoubensis*	CR	CR	Disease	CR(PE)
ANURA	PHRYNOBATRACHIDAE	*Phrynobatrachus njiomock*	CR	CR	Disease	CR(PE)
ANURA	PHRYNOBATRACHIDAE	*Phrynobatrachus steindachneri*	VU	VU	Disease	CR
ANURA	RANIDAE	*Lithobates miadis*	VU	VU	Reduced-habitat	CR
ANURA	RANIDAE	*Pelophylax caralitanus*	LC	NT	Reduced-habitat	VU
ANURA	RANIDAE	*Pelophylax chosenicus*	LC	NT	Reduced-habitat	VU
ANURA	RANIDAE	*Rana chevronta*	CR	CR	Reduced-habitat	CR(PE)
ANURA	RANIDAE	*Rana iberica*	LC	NT	Reduced-habitat	VU
ANURA	RANIDAE	*Rana pretiosa*	NT	NT	Climate change effects	VU
ANURA	RANIDAE	*Rana tavasensis*	EN	EN	Reduced-habitat	CR
ANURA	RANIDAE	*Rana tsushimensis*	LC	LC	Reduced-habitat	NT
ANURA	RHINODERMATIDAE	*Rhinoderma darwinii*	NT	VU	Reduced-habitat	EN
ANURA	SOOGLOSSIDAE	*Sechellophryne gardineri*	NT	NT	Climate change effects	EN
ANURA	SOOGLOSSIDAE	*Sechellophryne pipilodryas*	VU	VU	Climate change effects	CR
ANURA	SOOGLOSSIDAE	*Sooglossus sechellensis*	NT	NT	Climate change effects	EN
ANURA	SOOGLOSSIDAE	*Sooglossus thomasseti*	VU	VU	Climate change effects	CR
ANURA	TELMATOBIIDAE	*Telmatobius bolivianus*	VU	CR	Disease	CR(PE)
ANURA	TELMATOBIIDAE	*Telmatobius edaphonastes*	EN	CR	Disease	CR(PE)
ANURA	TELMATOBIIDAE	*Telmatobius espadai*	EN	CR	Disease	CR(PE)
ANURA	TELMATOBIIDAE	*Telmatobius hintoni*	LC	NT	Disease	VU
ANURA	TELMATOBIIDAE	*Telmatobius mendelsoni*	LC	VU	Disease	CR(PE)

Order	Family	Genus and species	1980 backcasted Red List category	2004 backcasted Red List category	Primary driver of change between 2004–2022	2022 GAA2 Red List category
ANURA	TELMATOBIIDAE	*Telmatobius sibiricus*	EN	CR	Disease	CR(PE)
CAUDATA	AMBYSTOMATIDAE	*Ambystoma cingulatum*	NT	VU	Climate change effects	EN
CAUDATA	AMBYSTOMATIDAE	*Ambystoma mexicanum*	EN	EN	Reduced-habitat	CR
CAUDATA	HYNOBIIDAE	*Hynobius geojeensis*	LC	LC	Reduced-habitat	EN
CAUDATA	HYNOBIIDAE	*Hynobius guttatus*	LC	LC	Reduced-habitat	NT
CAUDATA	HYNOBIIDAE	*Hynobius notialis*	NT	NT	Reduced-habitat	VU
CAUDATA	HYNOBIIDAE	*Hynobius quelpaertensis*	NT	NT	Reduced-habitat	VU
CAUDATA	HYNOBIIDAE	*Hynobius stejnegeri*	LC	LC	Climate change effects	NT
CAUDATA	HYNOBIIDAE	*Hynobius tsuensis*	LC	LC	Reduced-habitat	NT
CAUDATA	HYNOBIIDAE	*Hynobius unisacculus*	VU	VU	Reduced-habitat	EN
CAUDATA	PLETHODONTIDAE	*Batrachoseps gabrieli*	LC	LC	Climate change effects	NT
CAUDATA	PLETHODONTIDAE	*Batrachoseps incognitus*	NT	NT	Climate change effects	EN
CAUDATA	PLETHODONTIDAE	*Batrachoseps kawia*	LC	LC	Climate change effects	NT
CAUDATA	PLETHODONTIDAE	*Batrachoseps regius*	NT	NT	Climate change effects	EN
CAUDATA	PLETHODONTIDAE	*Batrachoseps robustus*	NT	NT	Climate change effects	EN
CAUDATA	PLETHODONTIDAE	*Batrachoseps stebbinsi*	VU	VU	Reduced-habitat	EN
CAUDATA	PLETHODONTIDAE	*Bolitoglossa chica*	NT	NT	Reduced-habitat	CR
CAUDATA	PLETHODONTIDAE	*Bolitoglossa indio*	LC	LC	Climate change effects	EN
CAUDATA	PLETHODONTIDAE	*Bolitoglossa insularis*	VU	VU	Climate change effects	CR
CAUDATA	PLETHODONTIDAE	*Bolitoglossa kamuk*	LC	LC	Climate change effects	VU
CAUDATA	PLETHODONTIDAE	*Bolitoglossa leandrae*	EN	EN	Reduced-habitat	CR
CAUDATA	PLETHODONTIDAE	*Bolitoglossa pygmaea*	LC	LC	Climate change effects	VU
CAUDATA	PLETHODONTIDAE	*Bolitoglossa robusta*	LC	LC	Reduced-habitat	VU
CAUDATA	PLETHODONTIDAE	*Bolitoglossa taylori*	NT	NT	Disease	EN
CAUDATA	PLETHODONTIDAE	*Dendrotriton sanctibarbarus*	VU	VU	Reduced-habitat	CR
CAUDATA	PLETHODONTIDAE	*Desmognathus imitator*	LC	LC	Climate change effects	NT
CAUDATA	PLETHODONTIDAE	*Desmognathus santeetlah*	LC	LC	Climate change effects	NT
CAUDATA	PLETHODONTIDAE	*Eurycea subfluvicola*	LC	LC	Climate change effects	VU
CAUDATA	PLETHODONTIDAE	*Hydromantes samweli*	LC	LC	Reduced-habitat	NT
CAUDATA	PLETHODONTIDAE	*Hydromantes shastae*	LC	LC	Reduced-habitat	NT
CAUDATA	PLETHODONTIDAE	*Hydromantes wintu*	LC	LC	Reduced-habitat	VU
CAUDATA	PLETHODONTIDAE	*Nototriton mime*	VU	VU	Reduced-habitat	CR
CAUDATA	PLETHODONTIDAE	*Nototriton picucha*	VU	VU	Reduced-habitat	CR
CAUDATA	PLETHODONTIDAE	*Nototriton saslaya*	VU	VU	Reduced-habitat	CR
CAUDATA	PLETHODONTIDAE	*Oedipina fortunensis*	NT	NT	Disease	EN
CAUDATA	PLETHODONTIDAE	*Plethodon sherando*	NT	NT	Climate change effects	VU

Order	Family	Genus and species	1980 backcasted Red List category	2004 backcasted Red List category	Primary driver of change between 2004–2022	2022 GAA2 Red List category
CAUDATA	PLETHODONTIDAE	*Speleomantes ambrosii*	NT	NT	Disease	CR
CAUDATA	PLETHODONTIDAE	*Speleomantes italicus*	NT	NT	Disease	EN
CAUDATA	PLETHODONTIDAE	*Speleomantes sarrabusensis*	VU	VU	Unknown	CR
CAUDATA	PLETHODONTIDAE	*Speleomantes strinatii*	NT	NT	Disease	EN
CAUDATA	PLETHODONTIDAE	*Thorius longicaudus*	EN	CR	Disease	CR(PE)
CAUDATA	RHYACOTRITONIDAE	*Rhyacotriton olympicus*	LC	LC	Climate change effects	NT
CAUDATA	SALAMANDRIDAE	*Calotriton arnoldi*	EN	EN	Disease	CR
CAUDATA	SALAMANDRIDAE	*Cynops pyrrhogaster*	LC	LC	Reduced-habitat	NT
CAUDATA	SALAMANDRIDAE	*Cynops yunnanensis*	LC	LC	Reduced-habitat	VU
CAUDATA	SALAMANDRIDAE	*Echinotriton chinhaiensis*	EN	EN	Reduced-habitat	CR
CAUDATA	SALAMANDRIDAE	*Ommatotriton ophryticus*	LC	LC	Reduced-habitat	NT
CAUDATA	SALAMANDRIDAE	*Salamandra lanzai*	VU	VU	Disease	CR
CAUDATA	SALAMANDRIDAE	*Salamandra salamandra*	LC	LC	Disease	VU
CAUDATA	SALAMANDRIDAE	*Salamandrina perspicillata*	LC	LC	Disease	EN
CAUDATA	SALAMANDRIDAE	*Triturus carnifex*	LC	LC	Disease	VU
CAUDATA	SALAMANDRIDAE	*Triturus marmoratus*	LC	LC	Disease	VU
CAUDATA	SALAMANDRIDAE	*Tylototriton shanjing*	LC	NT	Over-exploited	VU
CAUDATA	SALAMANDRIDAE	*Tylototriton taliangensis*	NT	NT	Over-exploited	VU
CAUDATA	SALAMANDRIDAE	*Tylototriton yangi*	VU	VU	Over-exploited	EN
GYMNOPHIONA	RHINATREMATIDAE	*Rhinatrema shiv*	LC	NT	Reduced-habitat	EN

Table 3b. Species that deteriorated in status between 2004–2022 categorized by primary drivers of decline.

Supplementary Table 4a

Order	Family	Genus and species	1980 backcasted Red List category	Primary threat mitigated between 1980–2004	Conservation action leading to improvement between 1980–2004	2004 backcasted Red List category	2022 GAA2 Red List category
ANURA	AROMOBATIDAE	*Allobates olfersioides*	VU	Disease	N/A	LC	LC
ANURA	BRACHYCEPHALIDAE	*Ischnocnema feioi*	EN	Timber and plant harvesting	Site/area protection	NT	NT
ANURA	BUFONIDAE	*Ansonia endauensis*	EN	Agriculture and aquaculture	Site/area protection	NT	NT
ANURA	BUFONIDAE	*Bufo ailaoanus*	EN	Timber and plant harvesting	Site/area protection	NT	NT
ANURA	BUFONIDAE	*Ghatophryne rubigina*	EN	Agriculture and aquaculture	Site/area protection	NT	NT
ANURA	CENTROLENIDAE	*Centrolene heloderma*	CR	Disease	N/A	VU	VU
ANURA	CERATOBATRACHIDAE	*Liurana alpina*	CR	Infrastructure development	Site/area protection	NT	NT
ANURA	ELEUTHERODACTYLIDAE	*Diasporus tigrillo*	CR	Agriculture and aquaculture	Site/area protection	NT	NT
ANURA	HYLIDAE	*Osteopilus vastus*	EN	Disease	N/A	VU	VU
ANURA	HYLIDAE	*Sarcohyla hazelae*	EN	Disease	N/A	VU	VU
ANURA	HYLODIDAE	*Hylodes japi*	EN	Agriculture and aquaculture	Site/area protection	NT	NT
ANURA	LEPTODACTYLIDAE	*Pleurodema bibroni*	EN	Disease	N/A	NT	LC
ANURA	LEPTODACTYLIDAE	*Pleurodema kriegi*	EN	Agriculture and aquaculture	Site/area protection	NT	NT
ANURA	MEGOPHRYIDAE	*Leptobrachella shangsiensis*	EN	Agriculture and aquaculture	Site/area protection	NT	NT
ANURA	MEGOPHRYIDAE	*Megophrys ombrophila*	CR	Infrastructure development	Site/area protection	NT	NT
ANURA	MEGOPHRYIDAE	*Megophrys wuliangshanensis*	VU	Timber and plant harvesting	Site/area protection	NT	NT
ANURA	MICRIXALIDAE	*Micrixalus kodayari*	CR	Agriculture and aquaculture	Site/area protection	NT	NT
ANURA	MICROHYLIDAE	*Glyphoglossus minutus*	EN	Agriculture and aquaculture	Site/area protection	NT	NT
ANURA	MYOBATRACHIDAE	*Mixophyes fleayi*	CR	Disease	N/A	EN	EN
ANURA	MYOBATRACHIDAE	*Mixophyes iteratus*	CR	Disease	N/A	VU	VU
ANURA	NYCTIBATRACHIDAE	*Nyctibatrachus vasanthi*	EN	Agriculture and aquaculture	Site/area protection	NT	NT
ANURA	RANIDAE	*Lithobates tarahumarae*	EN	Disease	N/A	VU	VU
ANURA	RHACOPHORIDAE	*Kurixalus lenquanensis*	EN	Agriculture and	Site/area protection	NT	NT

Order	Family	Genus and species	1980 backcasted Red List category	Primary threat mitigated between 1980–2004	Conservation action leading to improvement between 1980–2004	2004 backcasted Red List category	2022 GAA2 Red List category
				aquaculture			
ANURA	RHACOPHORIDAE	*Leptomantis gadingensis*	EN	Agriculture and aquaculture	Site/area protection	NT	NT
ANURA	RHACOPHORIDAE	*Raorchestes kakachi*	EN	Agriculture and aquaculture	Site/area protection	NT	NT
ANURA	RHACOPHORIDAE	*Raorchestes silentvalley*	EN	Agriculture and aquaculture	Site/area protection	NT	NT
ANURA	RHACOPHORIDAE	*Raorchestes uthamani*	EN	Timber and plant harvesting	Site/area protection	NT	NT
CAUDATA	HYNOBIIDAE	*Hynobius fucus*	EN	Infrastructure development	Site/area protection	NT	NT
CAUDATA	PLETHODONTIDAE	*Batrachoseps campi*	EN	Agriculture and aquaculture	Land/water management	NT	NT
CAUDATA	PLETHODONTIDAE	*Bolitoglossa diminuta*	CR	Agriculture and aquaculture	Site/area protection	NT	NT
CAUDATA	PLETHODONTIDAE	*Bolitoglossa sombra*	EN	Agriculture and aquaculture	Site/area protection	NT	NT
CAUDATA	PLETHODONTIDAE	*Chiropterotriton chico*	CR	Disease	N/A	VU	VU
CAUDATA	PLETHODONTIDAE	*Chiropterotriton dimidiatus*	CR	Disease	N/A	VU	VU
CAUDATA	PLETHODONTIDAE	*Thorius narisovalis*	CR	Disease	N/A	EN	EN
CAUDATA	PLETHODONTIDAE	Thorius pennatulus	CR	Disease	N/A	EN	EN

Table 4a. Species that improved in status between 1980–2004 categorized by primary threat mitigated. If applicable, the conservation action leading to the improvement in status is also included.

Supplementary Table 4b

Order	Family	Genus and species	1980 backcasted Red List category	2004 backcasted Red List category	2022 GAA2 Red List category	Primary threat mitigated between 2004–2022	Conservation action leading to improvement between 2004–2022
ANURA	BREVICIPITIDAE	*Breviceps macrops*	VU	VU	NT	Mining/Energy production	N/A
ANURA	BUFONIDAE	*Anaxyrus canorus*	VU	EN	VU	Disease	N/A
ANURA	BUFONIDAE	*Ansonia fuliginea*	VU	VU	NT	Timber and plant harvesting	Land/water management
ANURA	BUFONIDAE	*Ansonia platysoma*	VU	VU	NT	Timber and plant harvesting	Land/water management
ANURA	BUFONIDAE	*Atelopus elegans*	VU	CR	EN	Disease	N/A
ANURA	BUFONIDAE	*Atelopus exiguus*	EN	CR	EN	Disease	N/A
ANURA	BUFONIDAE	*Atelopus pulcher*	VU	CR	VU	Disease	N/A
ANURA	BUFONIDAE	*Ingerophrynus ledongensis*	VU	VU	NT	Agriculture and aquaculture	Site/area protection
ANURA	BUFONIDAE	*Peltophryne lemur*	EN	CR	EN	Infrastructure development	Reintroduction
ANURA	BUFONIDAE	*Rhinella leptoscelis*	LC	VU	NT	Disease	N/A
ANURA	BUFONIDAE	*Vandijkophrynus inyangae*	LC	EN	VU	Agriculture and aquaculture	Land/water management
ANURA	CENTROLENIDAE	*Centrolene ballux*	EN	CR	EN	Undetermined	N/A
ANURA	CENTROLENIDAE	*Centrolene medemi*	NT	CR	EN	Disease	N/A
ANURA	CENTROLENIDAE	*Rulyrana spiculata*	LC	VU	NT	Disease	N/A
ANURA	CERATOBATRACHIDAE	*Platymantis banahao*	VU	VU	NT	Human disturbance	Site/area protection
ANURA	CERATOBATRACHIDAE	*Platymantis indeprensus*	VU	VU	NT	Human disturbance	Site/area protection
ANURA	CERATOBATRACHIDAE	*Platymantis naomii*	VU	VU	NT	Human disturbance	Site/area protection
ANURA	CERATOBATRACHIDAE	*Platymantis pseudodorsalis*	VU	VU	NT	Human disturbance	Site/area protection
ANURA	CERATOPHRYIDAE	*Ceratophrys ornata*	VU	VU	NT	Agriculture and aquaculture	N/A
ANURA	CRAUGASTORIDAE	*Craugastor emleni*	EN	CR	EN	Disease	N/A
ANURA	CRAUGASTORIDAE	*Craugastor greggi*	EN	CR	EN	Disease	N/A
ANURA	CRAUGASTORIDAE	*Craugastor polymniae*	CR	CR	NT	Agriculture and aquaculture	Land/water management
ANURA	CRAUGASTORIDAE	*Craugastor sabrinus*	NT	EN	NT	Disease	N/A
ANURA	CRAUGASTORIDAE	*Craugastor taurus*	VU	CR	EN	Disease	N/A
ANURA	CRAUGASTORIDAE	*Pristimantis silverstonei*	VU	EN	VU	Disease	N/A
ANURA	CRAUGASTORIDAE	*Pristimantis tinajillas*	CR	CR	VU	Agriculture and aquaculture	Site/area protection

Order	Family	Genus and species	1980 backcasted Red List category	2004 backcasted Red List category	2022 GAA2 Red List category	Primary threat mitigated between 2004–2022	Conservation action leading to improvement between 2004–2022
ANURA	CYCLORAMPHIDAE	*Cycloramphus faustoi*	CR	CR	VU	Human disturbance	Site/area protection
ANURA	CYCLORAMPHIDAE	*Cycloramphus lithomimeticus*	CR	CR	NT	Infrastructure development	Site/area protection
ANURA	DENDROBATIDAE	*Andinobates dorisswansonae*	CR	CR	VU	Agriculture and aquaculture	Site/area protection
ANURA	DENDROBATIDAE	*Andinobates tolimensis*	CR	CR	VU	Agriculture and aquaculture	Site/area protection
ANURA	DENDROBATIDAE	*Hyloxalus elachyhistus*	LC	VU	LC	Disease	N/A
ANURA	DENDROBATIDAE	*Hyloxalus fascianigrus*	VU	CR	VU	Disease	N/A
ANURA	DENDROBATIDAE	*Hyloxalus pulchellus*	NT	VU	NT	Disease	N/A
ANURA	DENDROBATIDAE	*Hyloxalus vertebralis*	VU	CR	VU	Disease	N/A
ANURA	DICROGLOSSIDAE	*Nanorana unculuanus*	NT	EN	VU	Over-exploited	Compliance and enforcement
ANURA	ELEUTHERODACTYLIDAE	*Eleutherodactylus coqui*	LC	NT	LC	Disease	N/A
ANURA	ELEUTHERODACTYLIDAE	*Eleutherodactylus locustus*	EN	CR	EN	Disease	N/A
ANURA	ELEUTHERODACTYLIDAE	*Eleutherodactylus richmondi*	EN	CR	EN	Disease	N/A
ANURA	HEMIPHRACTIDAE	*Gastrotheca pseustes*	LC	EN	NT	Disease	N/A
ANURA	HEMIPHRACTIDAE	*Gastrotheca riobambae*	LC	EN	VU	Agriculture and aquaculture	Site/area protection
ANURA	HYLIDAE	*Bromeliohyla bromeliacia*	LC	EN	LC	Disease	N/A
ANURA	HYLIDAE	*Duellmanohyla ignicolor*	CR	CR	NT	Agriculture and aquaculture	Land/water management
ANURA	HYLIDAE	*Duellmanohyla uranochroa*	VU	CR	VU	Disease	N/A
ANURA	HYLIDAE	*Hyloscirtus armatus*	LC	VU	NT	Disease	N/A
ANURA	HYLIDAE	*Hyloscirtus colymba*	LC	CR	EN	Disease	N/A
ANURA	HYLIDAE	*Isthmohyla rivularis*	VU	CR	EN	Disease	N/A
ANURA	HYLIDAE	*Plectrohyla pokomchi*	EN	CR	EN	Disease	N/A
ANURA	HYLIDAE	*Sarcohyla celata*	EN	CR	NT	Disease	N/A
ANURA	HYLIDAE	*Sarcohyla thorectes*	EN	CR	EN	Disease	N/A
ANURA	HYLIDAE	*Scinax alcatraz*	CR	CR	VU	Human disturbance	Site/area protection
ANURA	HYLIDAE	*Scinax faivovichi*	CR	CR	VU	Infrastructure development	Site/area protection
ANURA	HYLIDAE	*Scinax feioi*	EN	EN	NT	Agriculture and aquaculture	Site/area protection
ANURA	HYLODIDAE	*Hylodes babax*	VU	VU	NT	Agriculture and aquaculture	Site/area protection
ANURA	LEIOPELMATIDAE	*Leiopelma hamiltoni*	EN	EN	VU	Invasive Species	Reintroduction
ANURA	LEPTODACTYLIDAE	*Pleurodema bibroni*	EN	NT	LC	Disease	N/A
ANURA	LIMNODYNASTIDAE	*Adelotus brevis*	LC	NT	LC	Disease	N/A

Order	Family	Genus and species	1980 backcasted Red List category	2004 backcasted Red List category	2022 GAA2 Red List category	Primary threat mitigated between 2004–2022	Conservation action leading to improvement between 2004–2022
ANURA	MEGOPHRYIDAE	*Leptobrachella arayai*	VU	VU	NT	Timber and plant harvesting	Land/water management
ANURA	MEGOPHRYIDAE	*Leptobrachella maura*	VU	VU	NT	Timber and plant harvesting	Land/water management
ANURA	MEGOPHRYIDAE	*Leptobrachium gunungense*	VU	VU	NT	Timber and plant harvesting	Land/water management
ANURA	MEGOPHRYIDAE	*Megophrys baluensis*	VU	VU	NT	Timber and plant harvesting	Land/water management
ANURA	MEGOPHRYIDAE	*Megophrys dringi*	VU	VU	NT	Timber and plant harvesting	Land/water management
ANURA	MEGOPHRYIDAE	*Megophrys feii*	CR	CR	NT	Agriculture and aquaculture	Land/water management
ANURA	MICROHYLIDAE	*Kalophrynus baluensis*	VU	VU	NT	Timber and plant harvesting	Land/water management
ANURA	MYOBATRACHIDAE	*Pseudophryne bibronii*	LC	NT	LC	Disease	N/A
ANURA	ODONTOPHRYNIDAE	*Proceratophrys rotundipalpebra*	EN	EN	NT	Agriculture and aquaculture	Site/area protection
ANURA	PELODRYADIDAE	*Litoria aurea*	NT	VU	NT	Disease	N/A
ANURA	PELODRYADIDAE	*Litoria dayi*	LC	EN	VU	Disease	N/A
ANURA	PELODRYADIDAE	*Litoria nannotis*	LC	EN	LC	Disease	N/A
ANURA	PELODRYADIDAE	*Litoria pearsoniana*	LC	NT	LC	Disease	N/A
ANURA	PELODRYADIDAE	*Litoria raniformis*	EN	EN	VU	Disease	N/A
ANURA	PELODRYADIDAE	*Litoria rheocola*	LC	EN	NT	Disease	N/A
ANURA	PHYLLOMEDUSIDAE	*Agalychnis annae*	NT	EN	VU	Disease	N/A
ANURA	RANIDAE	*Amolops daiyunensis*	EN	EN	NT	Agriculture and aquaculture	Land/water management
ANURA	RANIDAE	*Amolops yunkaiensis*	CR	CR	NT	Infrastructure development	Site/area protection
ANURA	RANIDAE	*Lithobates vibicarius*	VU	CR	EN	Disease	N/A
ANURA	RANIDAE	*Meristogenys stenocephalus*	EN	EN	NT	Timber and plant harvesting	Land/water management
ANURA	RANIDAE	*Meristogenys stigmachilus*	EN	EN	NT	Timber and plant harvesting	Land/water management
ANURA	RHACOPHORIDAE	*Raorchestes honnametti*	EN	EN	NT	Agriculture and aquaculture	Site/area protection
ANURA	RHACOPHORIDAE	*Raorchestes indigo*	CR	CR	VU	Mining/Energy production	Compliance and enforcement

Order	Family	Genus and species	1980 backcasted Red List category	2004 backcasted Red List category	2022 GAA2 Red List category	Primary threat mitigated between 2004–2022	Conservation action leading to improvement between 2004–2022
ANURA	RHACOPHORIDAE	*Zhangixalus minimus*	VU	VU	NT	Timber and plant harvesting	Land/water management
ANURA	RHACOPHORIDAE	*Zhangixalus yaoshanensis*	EN	EN	NT	Timber and plant harvesting	N/A
CAUDATA	PLETHODONTIDAE	*Bolitoglossa pesrubra*	LC	VU	LC	Undetermined	N/A
CAUDATA	PLETHODONTIDAE	*Chiropterotriton magnipes*	EN	CR	EN	Disease	N/A
CAUDATA	PLETHODONTIDAE	*Nototriton picadoi*	NT	NT	LC	Agriculture and aquaculture	Site/area protection
CAUDATA	PLETHODONTIDAE	*Nototriton richardi*	NT	NT	LC	Agriculture and aquaculture	Site/area protection
CAUDATA	PLETHODONTIDAE	*Oedipina uniformis*	LC	NT	LC	Undetermined	N/A
CAUDATA	PLETHODONTIDAE	*Pseudoeurycea rex*	LC	CR	VU	Disease	N/A
CAUDATA	PLETHODONTIDAE	*Thorius adelos*	CR	CR	NT	Agriculture and aquaculture	Land/water management

Table 4b. Species that improved in status between 2004–2022 categorized by primary threat mitigated. If applicable, the conservation action leading to the improvement in status is also included.
