# Supplementary material for: Ongoing declines for the world’s amphibians in the face of emerging threats
Source: Nature. 2023 Oct 4;622(7982):308–14. doi: 10.1038/s41586-023-06578-4 (PMC10567568; doi:10.1038/s41586-023-06578-4)
Supplement: Supplementary file 2 — Reporting Summary [file 41586_2023_6578_MOESM2_ESM.pdf]

nature portfolio

Corresponding author(s): Jennifer A. Luedtke

Last updated by author(s): 30 June 2023

Reporting Summary

Nature Portfolio wishes to improve the reproducibility of the work that we publish. This form provides structure for consistency and transparency in reporting. For further information on Nature Portfolio policies, see our Editorial Policies and the Editorial Policy Checklist.

Statistics

For all statistical analyses, confirm that the following items are present in the figure legend, table legend, main text, or Methods section.

n/a	Confirmed	
☐	☒	The exact sample size (*n*) for each experimental group/condition, given as a discrete number and unit of measurement
☒	☐	A statement on whether measurements were taken from distinct samples or whether the same sample was measured repeatedly
☒	☐	The statistical test(s) used AND whether they are one- or two-sided<br>*Only common tests should be described solely by name; describe more complex techniques in the Methods section.*
☒	☐	A description of all covariates tested
☒	☐	A description of any assumptions or corrections, such as tests of normality and adjustment for multiple comparisons
☐	☒	A full description of the statistical parameters including central tendency (e.g. means) or other basic estimates (e.g. regression coefficient) AND variation (e.g. standard deviation) or associated estimates of uncertainty (e.g. confidence intervals)
☒	☐	For null hypothesis testing, the test statistic (e.g. *F*, *t*, *r*) with confidence intervals, effect sizes, degrees of freedom and *P* value noted<br>*Give P values as exact values whenever suitable.*
☒	☐	For Bayesian analysis, information on the choice of priors and Markov chain Monte Carlo settings
☒	☐	For hierarchical and complex designs, identification of the appropriate level for tests and full reporting of outcomes
☒	☐	Estimates of effect sizes (e.g. Cohen's *d*, Pearson's *r*), indicating how they were calculated

Our web collection on statistics for biologists contains articles on many of the points above.

Software and code

Policy information about availability of computer code

Data collection	No software was used.
Data analysis	No software was used.

For manuscripts utilizing custom algorithms or software that are central to the research but not yet described in published literature, software must be made available to editors and reviewers. We strongly encourage code deposition in a community repository (e.g. GitHub). See the Nature Portfolio guidelines for submitting code & software for further information.

Data

Policy information about availability of data

All manuscripts must include a data availability statement. This statement should provide the following information, where applicable:

- Accession codes, unique identifiers, or web links for publicly available datasets
- A description of any restrictions on data availability
- For clinical datasets or third party data, please ensure that the statement adheres to our policy

The spatial and raw tabular data analysed in this study are available at https://doi.org/10.5061/dryad.xgxd254n5.
The GAA2 IUCN Red List assessments, including range maps, for all 8,011 species will also be available for download on The IUCN Red List of Threatened SpeciesTM website (https://iucnredlist.org) following its September 2023 update (version 2023–1).

In rare cases, a species may be threatened because of over-collection and sensitive distribution information is not publicly available.
Source data are provided with this paper.

Research involving human participants, their data, or biological material

Policy information about studies with human participants or human data. See also policy information about sex, gender (identity/presentation), and sexual orientation and race, ethnicity and racism.

Reporting on sex and gender	N/A
Reporting on race, ethnicity, or other socially relevant groupings	N/A
Population characteristics	N/A
Recruitment	N/A
Ethics oversight	N/A

Note that full information on the approval of the study protocol must also be provided in the manuscript.

Field-specific reporting

Please select the one below that is the best fit for your research. If you are not sure, read the appropriate sections before making your selection.

☐ Life sciences ☐ Behavioural & social sciences ☒ Ecological, evolutionary & environmental sciences

For a reference copy of the document with all sections, see nature.com/documents/nr-reporting-summary-flat.pdf

Ecological, evolutionary & environmental sciences study design

All studies must disclose on these points even when the disclosure is negative.

Study description	This study examines the 8,011 amphibian species with an extinction risk assessment for the IUCN Red List of Threatened Species. Trends in extinction risk are quantified for 1980, 2004, and 2022 with comparisons between species in the different biogeographic realms, taxonomic orders, and breeding strategies. Estimates of extinction risk using current data are made for the species that were not known to science in 1980 and 2004. A particular focus of the study is the drivers of genuine extinction risk changes as these reflect actual increases or decreases in threat levels, some due to targeted conservation actions. These results are relevant to global, national, and local conservation planning and prioritisation, the National Biodiversity Action Plans (NBSAPs) reported to the Convention on Biological Diversity (CBD) of the United Nations to track progress towards the Kunming-Montreal Global Biodiversiy Framework adopted by 190+ signatory countries at COP15 in Montreal, Canada in December 2022.
Research sample	The sample size of this study includes 8,011 amphibian species known to science, representing 92.9% of described amphibians on the 3 May 2023 resubmission date.
Sampling strategy	The entire sample was used.
Data collection	Raw data collection took place between 2012-2022 resulting in IUCN Red List categories and their accompanying information for each species. This information comprises one of the two datasets in this study. This process involved more than 1,000 subject-matter experts through the consultation process described in the Methods section of the manuscript. Backcasting of the categories took place in 2022, which comprises the second dataset analysed in this study.
Timing and spatial scale	Data collection took place between 2012-2022. The data cover the taxonomy and geographic range of the 8,011 amphibian species in this study, i.e. every continent except Antarctica.
Data exclusions	No data were excluded.
Reproducibility	The data made available in the manuscript, Supplementary Information, and the data repository linked above enable the reproduction of all analyses and results.
Randomization	No randomisation was necessary for the analyses inf this study.
Blinding	Blinding was not relevant to this study.

Did the study involve field work? ☐ Yes ☒ No

Reporting for specific materials, systems and methods

We require information from authors about some types of materials, experimental systems and methods used in many studies. Here, indicate whether each material, system or method listed is relevant to your study. If you are not sure if a list item applies to your research, read the appropriate section before selecting a response.

Materials & experimental systems

n/a	Involved in the study
☒	☐ Antibodies
☒	☐ Eukaryotic cell lines
☒	☐ Palaeontology and archaeology
☒	☐ Animals and other organisms
☒	☐ Clinical data
☒	☐ Dual use research of concern
☒	☐ Plants

Methods

n/a	Involved in the study
☒	☐ ChIP-seq
☒	☐ Flow cytometry
☒	☐ MRI-based neuroimaging
